# The McNorm library: creating and validating a new library of emotionally expressive whole body dance movements

**DOI:** 10.1007/s00426-022-01669-9

**Published:** 2022-04-06

**Authors:** Rebecca A. Smith, Emily S. Cross

**Affiliations:** 1 Institute of Neuroscience and Psychology, University of Glasgow, Glasgow, Scotland; 2 Department of Cognitive Science, Macquarie University, Sydney, Australia

## Abstract

The ability to exchange affective cues with others plays a key role in our ability to create and maintain meaningful social relationships. We express our emotions through a variety of socially salient cues, including facial expressions, the voice, and body movement. While significant advances have been made in our understanding of verbal and facial communication, to date, understanding of the role played by human body movement in our social interactions remains incomplete. To this end, here we describe the creation and validation of a new set of emotionally expressive whole-body dance movement stimuli, named the Motion Capture Norming (McNorm) Library, which was designed to reconcile a number of limitations associated with previous movement stimuli. This library comprises a series of point-light representations of a dancer’s movements, which were performed to communicate to observers neutrality, happiness, sadness, anger, and fear. Based on results from two validation experiments, participants could reliably discriminate the intended emotion expressed in the clips in this stimulus set, with accuracy rates up to 60% (chance = 20%). We further explored the impact of dance experience and trait empathy on emotion recognition and found that neither significantly impacted emotion discrimination. As all materials for presenting and analysing this movement library are openly available, we hope this resource will aid other researchers in further exploration of affective communication expressed by human bodily movement.

**Supplementary Information:**

The online version contains supplementary material available at 10.1007/s00426-022-01669-9.

The ability to communicate with others plays a vital role in our ability to successfully navigate our social world. Successful interactions with others have a substantial impact on how we develop and maintain meaningful relationships, and also have consequences for our sense of belonging to a wider social community, as well as our general mental well-being (Caplan, 2003; Shankar et al., 2015). These interactions are multi-faceted, and researchers have made substantial advances in our understanding of how facial expressions (Du et al., 2014; Jack et al., 2014; Jack et al., 2012a; Jack et al., 2012b) and verbal communication (McAleer et al., 2014; Whiting et al., 2020) mediate the success of our interactions with others. However, key elements of non-verbal communication have been somewhat neglected in this area of research. Human body movement, for example, provides a rich source of information, whose social value to observers is only beginning to be explored (Williams & Cross, 2018; Williams et al., 2019). In fact, emerging research suggests that the human body may provide even more salient cues to emotion than the face (Aviezer et al., 2012a, 2012b; Wang et al., 2018). At present, however, no framework exists that attempts to explain the role played by human body motion in our social interactions. Until this field receives more dedicated research attention, our appreciation of human social signalling, in all of its complexities, will remain incomplete.

A number of movement libraries have been created to explore what observers can identify from simple, everyday human motions (Dekeyser et al., 2002; Ma et al., 2006; Vanrie & Verfaillie, 2004). These libraries vary greatly in the type and complexity of movements they capture, with some focusing on walking motions and others exploring the movement of isolated body areas (e.g., simple arm movements like pointing, waving, and grasping). They also vary in the way visual information about the body is presented, with some depicting movement in the form of full video recordings and others depicting the human form with a reduction of surface-level visual information about a person’s form; for example, by rendering human movement as Point-Light Displays (PLDs). PLDs were originally developed by Johansson when he observed that the kinematics of biological motion could be depicted by attaching small light sources to the major joints of a model’s body (Johansson, 1975; Krüger et al., 2018). The output of these displays are dot configurations that, when animated, leave the viewer with the impression that they are watching a person (or other animate being) in motion. This introduced the idea that the visual system can interpret animate motion from abstract representations of human figures which are devoid of form cues and other superficial visual information (Chang et al., 2018). Even without morphological cues, research has shown that humans are able to identify a range of features from PLD motion. For example, it has been found that people are highly accurate at identifying the gender (with up to 71% accuracy; see review by Pollick et al., 2005) and identity of PLD walkers (Mitchell & Curry, 2016), and can attribute higher order social constructs like personality traits and sexuality to these abstract figures and dot configurations (Heberlein et al., 2004; Johnson et al., 2007). In addition, it has been found that individuals can rapidly and reliably attribute affective states to PLD representations of gait cycles and everyday actions (Atkinson et al., 2004; Gunns et al., 2002; Heberlein et al., 2004; Schneider et al., 2014).

PLDs are extremely useful in human movement and affective science research. In emotion research that aims to evaluate the contribution of one particular expressive cue, it is standard practice to remove extraneous contextual information from stimuli. In movement libraries, auditory cues (e.g., music, and exertion sounds like breathing and gasping) are typically removed (Jola et al., 2014, are a notable exception), and the performer’s face is blurred to remove the competing influence of facial expressions on social judgements (Christensen et al., 2014; Melzer et al., 2019). PLDs represent an extension of this rationale, and as such are a uniquely useful tool for exploring pure motion, in isolation from all other emotionally salient communicative cues (e.g., non-movement related visual cues like facial expressions, and the appearance of the dancer). A number of PLD movement libraries have been created to further the study of human emotion recognition from body movement.

While these stimuli libraries are inarguably useful for exploring how everyday human movements communicate emotional expression to observers, they also have several limitations worth considering. The first is that many of these stimuli depict emotion through pantomimed actions (e.g., shaking fist in anger). While humans do use these kinds of cues to extract high-level social information from others in the real world, we are also able to infer this information from far more nuanced demonstrations of authentic expression. Including these iconic gestural cues in movement sequences likely obscures the influence of subtler components of human movement that provide expressive information. The inclusion of more literal or iconic gestures in libraries may also raise questions about the content validity of studies using them to explore the relationship between motion and emotion recognition, as it could be argued that participants in these studies are providing measures of their ability to successfully recognise social gestures, rather than their ability to infer expression from the performative elements of movement.

Second, it has already been noted that these libraries vary greatly in their content and the way they represent the human form. The issue with the scope and variability of these libraries is that this presents significant difficulties for comparing the results across studies and for extracting the most salient results. In the case of exploring emotion recognition from human movement specifically, this has given rise to large inconsistencies in the reported recognition rates for different emotion categories. Often the range of emotions explored in these libraries is very narrow, with authors choosing to focus on recognition of only one or two basic emotions or only on the distinction between positive and negative emotional valence (Castellano et al, 2007; Michalak et al., 2009; Huis in ‘t Veld et al., 2014a, Huis in ‘t Veld et al., 2014b). This ignores the diverse range of emotions each of these categories encompass: in essence, equating more complex emotions like anger or fear with sadness. Alternatively, the scope of emotions used in other works may be too broad; with some studies including portrayals of more than 10 specific emotions (Paterson et al., 2001; Walbott, 1998), and exploring more abstract concepts which are sensitive to cultural or interpretational variance (e.g., pride, shame, strength). However, even studies which account for the middle ground between these approaches (those which focus on several basic emotions, e.g., happiness, sadness, anger, and fear) report inconsistent recognition rates. Successful recognition of happiness, sadness, anger, and fear have been found to vary from 23–92% depending on the specific methodologies of each study, and recognition of neutral emotional displays (i.e., the absence of any clear emotional expression) also vary widely (Atkinson et al., 2004; Crane & Gross, 2013; Dael et al., 2012; Gross et al., 2010; Roether et al., 2009). A brief overview of average recognition rates obtained in response to various types of movement libraries reported in previous works can be found in Table 1 of the supplementary materials (Alaerts et al., 2011; Atkinson et al., 2004; Atkinson et al., 2007; Bachmann et al., 2020; Bernhardt & Robinson, 2007; Camurri et al., 2003; Christensen et al., 2016; Christensen et al., 2019; Christensen et al., 2021; Crane & Gross, 2013; Dael et al., 2012; Dahl & Friberg, 2007; Dittrich et al., 1996; Grezes et al., 2007; Gross et al., 2010; Gross et al., 2012; Melzer et al., 2019; Montepare et al., 1999; Pasch & Poppe, 2007; Roether et al., 2009).

**Table 1:** Demographics information for participants McNorm Experiment 1 (*N* = 50)

Age	Mean (SD)	29.06 (11.8)
	Range	18–63
Gender	Female	32
	Male	15
	Transgender female	1
	Transgender male	2
	Gender variant/non-conforming	NA
Dance level	Non-dancer	13
(self-reported)	Beginner	16
	Intermediate	15
	Advanced	4
	Professional	2

To begin to address these problems, greater cohesion (or at least clearer correspondence) between different researchers’ methodological approaches would help tremendously for building a more reliable and generalisable evidence base on the relationship between body movement and emotional expression. One of the most prominent issues to address in future movement libraries is the inclusion of socially relevant gestures in motion sequences. This is important because gestural communication is highly sensitive to cultural nuance. For example, in many Western countries (e.g., UK, USA) a ‘thumbs up’ is a positive symbol (indicating something is good) or is used to indicate that you are looking to share a car ride, but in other countries (e.g. Iran) this gesture is an insult, meaning something akin to “up yours” (Archer, 1997). It is likely that being presented with a positive symbol, or an obscenity (depending on cultural background) will influence a participant’s emotional state. Therefore, movement libraries which include instances of gestural communication may be unsuitable for research to explore more universal features of emotion recognition, particularly when using culturally diverse samples. To overcome such issues, it would be useful to ensure movement sequences feature movements that are not culturally specific, nor inherently tied to any particular emotional state. This should allow researchers to explore the impact of more nuanced movement features, and the expressive quality of motion on perceptions of emotion in observers. One way to reduce the impact of contextual cues on recognition is to use more abstract movements, rather than everyday motions, when creating stimuli libraries. Recently, it has been noted that dance can be of great value to social communication research in general (Orgs et al., 2018; Van Dyck et al., 2012), and to emotion research in particular (Aristidou et al., 2017; Van Dyck et al., 2017). Dance is, at its heart, the purposeful performance of expressive whole-body movements designed to communicate a narrative or meaning to observers. The inherently communicative nature of dance and the flexibility of its design (at both the choreographic and performative level) make dance ideally suited for the exploration of emotion expression by the human body in motion.

Christensen and colleagues have created two such dance libraries that have been used in emotion research. For their first library, they took 203 movement taken from recordings of full ballet performances, and blurred the dancers’ faces to remove the impact of facial cues (Christensen et al., 2014). These stimuli were evaluated for 25 movement characteristics, and were also rated for affective valence, arousal, and aesthetic appeal. The choreography was also annotated to provide detail about the specific movements contained in each sequence. Libraries like this (full light displays depicting the dancer in full costume, with set backdrops performing on stage) are particularly useful for aesthetic researchers, as these stimuli are more representative of authentic performative dance than others which contain reduced visual detail (e.g., PLDs). However, for use in emotion research, several critical limitations are associated with this type of stimuli. Using recordings of live dance performances, which contain a variety of visual confounds, may influence emotion judgements, particularly in a dance-experienced sample. It is extremely likely that experienced dancers will recognise elements of choreography from these stimuli and will have a number of these movements in their own repertoire. Familiarity with and intimate knowledge of such movement sequences may influence emotional responses and interfere with recognition data. In addition, costumes or stage furniture may provide cues about the narrative the dance is conveying, which even inexperienced observers can use to infer the emotional content, rather than simply relying on the movements themselves.

A more general issue in this area stems from many of these dance libraries assigning movements into emotion categories based solely on subjective perceptions made by observers, with no consideration given to the intention of the mover while the movement was being performed. By excluding intentionality from evaluating the components of expressive movement, this undermines the dyadic nature of authentic human interaction (Orgs et al., 2016). This issue was addressed in a more recent movement library created by Christensen and colleagues (2019). The Warburg Dance Movement (WADAMO) Library contains a series of movement sequences which were performed three times, each time with a different expressive intention (non-expressive, expressive-positive, or expressive-negative). These stimuli were then shown to dancers and dance-naïve observers, and the intended expression was compared to the expression perceived by observers (Christensen et al., 2019). The WADAMO library represents a substantial improvement on previous work by accounting for both the intention of the performer and the perception of the observer. Together, this makes for more robust classification of stimuli into different expressive categories. However, this library only accounts for the relationship between human motion and expressive valence, rather than providing information about the relationship between movement and the perception of specific emotions. Without creating libraries of this type, ones which address both sides of the interaction dyad and explore the expression of specific emotional states without the inclusion of overt gestures, our understanding of how body movement contributes to human expressivity will remain extremely limited. The Motion Capture Norming (McNorm) library developed in the present study aims to address these important gaps in the research.

## The McNorm library

### Description

The Motion Capture Norming (McNorm) library contains a series of whole-body ballet and contemporary dance movement sequences depicted in the form of PLDs. These sequences were new, original pieces of choreography that were devised and performed by a professional dancer. The full library (at the time of recording) comprised 17 different dance sequences. Each sequence was performed five times, each time with the aim to communicate a different emotion to observers (neutral, happy, sad, angry, and fearful) while maintaining the same choreography across each performance of the same sequence. Therefore, at the time of recording, the McNorm library comprised 85 recordings (17 of each emotion category). Technical issues, and missing data from the recording phase meant two of these sequences (2 sequences × 5 emotion portrayals = 10 recordings), and 2 individual recordings (1 angry, and 1 fearful) could not be included in the final library validated in this experiment. Therefore, this validation experiment was conducted on 73 emotionally expressive dance movement sequences (15 neutral, 15 happy, 15 sad, 14 angry, and 14 fearful).

### Rectifying limitations of previous movement libraries for use in emotion recognition studies

It has already been noted that many pre-existing movement libraries rely on the emotion judgements of observers to assign expressive movement into different emotion categories. However, the subjective nature of this task creates issues for ensuring the movements assigned to different emotion categories are truly representative of that category. To rectify this issue, the McNorm library was created with expressive intention of the performer in mind. In the validation of this library, the intended emotion of the performer is compared with the subjective emotion categorisation decisions of observers. If intention and subjective judgements align in this validation study, then the McNorm library has generated a more representative sample of expressive motions; one that more effectively captures the dyadic nature of human body movement in communication (between the movement performer and movement observer).

Further, the McNorm library depicts the dynamic human body in the form of PLDs. While limitations are associated with reducing the human form to a configuration of moving dots, this serves to limit the influence of superficial visual cues on emotion recognition. This approach ensures that researchers can use the McNorm library to study motion in isolation from other visual and social cues that are salient to all observers (e.g., perceptions of facial expressions, attractiveness and race of the movement performer) and cues which are of particular relevance to dance-experienced observers (e.g., costumes, stage furniture, etc.).

### Aims and predictions of the validation study

Our central aim with this validation study is to determine whether the performed emotion (i.e., the emotion intended on the dancer’s part) was reliably perceived by observers. As such, we hypothesised that the intended emotion would be recognised at greater than chance level by the participants. Based on the previous literature (see Table 1 in the supplementary materials for an overview of previous work in this field), we also predicted differences in recognition rates across the different emotion categories. Fear is not frequently explored in relation to this research question, but recognition rates which have been reported tend to be relatively low. Therefore, it is likely that fear will be recognised with lowest accuracy, in comparison with the other emotions explored in this study (Atkinson et al., 2007; Camurri et al., 2003; Dahl & Friberg, 2007; Dittrich et al., 1996; Pasch & Poppe, 2007).

Another aim of this study was to explore the impact of dance experience and trait empathy on emotion recognition capabilities. Dance experience has been found to impact a number of neuropsychological and behavioural outcomes (including emotion recognition capabilities; for a review, see Bläsing et al., 2012). For example, it has been shown that dance training results in significant changes to activity and organisation of sensorimotor structures that compose the action observation network (AON) in the human brain. Several studies have shown that professional dancers show greater engagement within the AON when watching dance compared to non-dancers, and this activity is amplified when dancers observe movement styles they have extensive physical experience performing (Calvo-Merino et al., 2005; Cross et al., 2006). In addition, even purely visual experience with dance has been shown to shape AON responses (Cross et al., 2009; Kirsch & Cross, 2015). There is also evidence of synaptic pruning in subcortical structures associated with the AON and more symmetrical activation of relevant occipitotemporal regions, as a direct result of dance training, which have been argued to reflect more efficient communication between areas involved in this complex neural circuit (Hänggi et al., 2010; Orlandi & Proverbio, 2019).

Beyond neuroimaging studies, it has been shown that dance experience can impact how individuals perceive and respond to the human body in motion. Results from Stevens and colleagues (2010) suggest that dancers and choreographers may develop specific visual-search patterns which influence visual attention when observing complex dance movements, and that these are driven by their movement expertise and learned experiences (Stevens et al., 2010). Studies have also noted that dancers appear to recognise affective expression from human movement with greater accuracy than individuals with no prior dance experience. In one such study, 24 non-dancers and 19 expert ballet dancers observed a series of 5–6 s ballet sequences and provided valence ratings on a slider scale from *very sad* (0) to *very happy* (100). It was found that dancers were significantly more accurate in their emotion classifications for recognition of positive affect than non-dancers (Christensen et al., 2016). Similar results were observed in a later study, wherein participants from a variety of dance backgrounds were asked to observe a series of movement sequences and identify whether these movements were expressive or non-expressive (Christensen et al., 2019). The authors report that years of dance experience correlated with accuracy in recognition of the intended expression, providing further support for the idea that dance experience facilitates the ability to recognise expression from emotion whole-body movement displays. Therefore, in addition to the emotion recognition task, participants will be asked to complete a shortened version of the Gold-DSI (Rose et al., 2020) to provide detail about their physical and observational engagement with dance. These factors will be explored in relation to emotion recognition accuracy to determine whether different levels of dance experience mediate the ability to interpret expressive cues from the movements of others.

Individual differences in empathy were also identified as another relevant factor to explore. Many previous studies have observed a link between empathy and the ability to successfully interpret emotional cues presented by others in our social environment. This has been observed across emotion recognition tasks using a variety of social cues; including faces, voices, body postures, and body movements (Balconi & Bortolotti, 2012; Besel & Yuille, 2010; Holland et al., 2021; Israelashvili et al., 2020; Jospe et al., 2018; Neumann et al., 2014; Rizzolatti & Craighero, 2005; Soto & Levenson, 2009). This link is even clearer when considering Autism Spectrum Disorders (ASD). Individuals with ASD tend to provide lower self-reported levels of empathy, and typically show lower performance on empathy-related tasks compared to typically developed individuals (Bishop & Seltzer, 2012; Demurie et al., 2011; Kok et al., 2016; Pepper et al., 2019; Trimmer et al., 2017). Furthermore, a common characteristic of ASD is difficulty in interpreting social cues, including the expression of emotions. Considering these factors together, it is plausible to expect that individual differences in empathy may mediate the ability to successfully interpret emotion expression from social behaviours.

Furthermore, research suggests there may also be a link between movement expertise and empathy, and these factors may interact to produce changes in emotion recognition capabilities. For example, it has been found that dancers have higher interoceptive accuracy than individuals without such movement training (Christensen et al., 2018), and more skilled dancers provide higher self-reported levels of emotional intelligence (Petrides et al., 2006). While these studies do not provide a direct link between movement expertise and increased empathy, the increasing implementation of dance movement therapy in the management of ASD suggests that dance may facilitate the development of empathic behaviours (Behrends et al., 2016; McGarry & Russo, 2011; Federman, 2011; Koch et al., 2015; Mastrominico et al., 2018). To this end, participants in the McNorm experiments will also complete the Toronto Empathy Scale (Spreng et al., 2009), to generate individual trait empathy scores for consideration in the analysis.

## Methods

### MCNORM library creation

#### Participants

One female professional dancer, previously a principal dancer with the Scottish Ballet, participated in the stimuli creation procedure. The dancer was chosen due to her extensive training, totalling more than 9 years with professional companies and 4 years in a freelance capacity, as well as her additional choreographic experience. The dancer contributed over a 6-day period (2 days to generate choreography and a 4-day recording period) and was provided with an honorarium for her time.

#### Stimuli creation

Movement sequences were recorded in the University of Glasgow’s motion capture lab, using 12 Vicon MXF40 cameras, recording at a rate of 120 frames per second (120 fps), to produce point-light displays for a new library of expressive dance movements.

##### Creation and recording of movement sequences

In advance of the filming dates, the dancer was asked to create a series of classical and contemporary ballet sequences. The dancer was informed that these movement sequences should contain only neutral movements (i.e., movements not inherently linked to a particular narrative, or emotional expression), to limit interference from previously acquired knowledge when performing the choreography. The dancer was also instructed that these movement sequences should have a minimum duration of 6 s. No maximum duration was explicitly defined, but the dancer was informed that longer sequences may be prone to more technical errors during recording and that she should not create sequences that, when performed repeatedly, would cause unnecessary fatigue.

The dancer was informed, prior to recording days, that the objective would be to perform each movement sequence five times, each time maintaining the same choreography but portraying a different emotional state; neutral (non-expressive), happy, sad, angry, and fearful. The experimenter did not show any movements, nor did she give examples or directions regarding expression; therefore, these expressive portrayals reflect the dancer’s personal interpretation of the different emotional states. For the neutral performances, the dancer was instructed to perform the movements with the same technical accuracy (with the same level of mechanical precision) but without expressivity; as if she was “going through the motions” or “working through an exercise”. To further limit interference from the experimenter, all communication about expression and technicalities of the sequences were discussed verbally, rather than through potentially leading gestures or body movement. Sequences were re-recorded at the dancer’s request when she had made a misstep or felt that she had not portrayed the emotion in a satisfactory way, and only at the experimenter’s request when there was a technical issue. Finally, while each sequence had a different musical accompaniment (to aid with timing, recall of the choreography and the feeling of performance of these movements), the music remained constant across all emotion portrayals, and all audio was removed as part of the final stimuli set.

The first day of the four-day filming period served as a trial run. The dancer spent 30 min at the start of each day warming up to ensure she would not injure herself, before practicing the choreography. The dancer then performed each movement sequence, always starting with the neutral portrayal before moving on to the other expressive categories in an order selected by the dancer (to ensure the dancer was in the correct mindset and the resultant expression felt authentic).

During the filming stage, 39 retroreflective markers were placed on the dancer’s body in anatomical regions defined by the Plug-in Gait Model, which is widely accepted for use in biological motion research (Kainz et al., 2017; Piwek et al., 2016). This placement was checked before and after each recording to ensure no markers had come loose or had fallen off and corrected where necessary.

In review, 17 sequences were performed, with each sequence performed five times (neutral, happy, sad, angry, fearful), creating a total of 85 recordings for the first iteration of this stimulus library. These recordings ranged in duration from 6.6 to 42.8 s, with an average duration of 22.1 s. Sad clips, on average were the longest in duration (average = 24.3 s), while angry clips were the shortest in duration (average = 20.6 s). The average duration of neutral, happy, and fearful clips were 21.1, 21.9, and 22.5 s respectively. Further detail about each of the stimuli recordings can be found in Supplementary Tables 3 and 4.

##### Point-light display creation

**Fig. 1:**
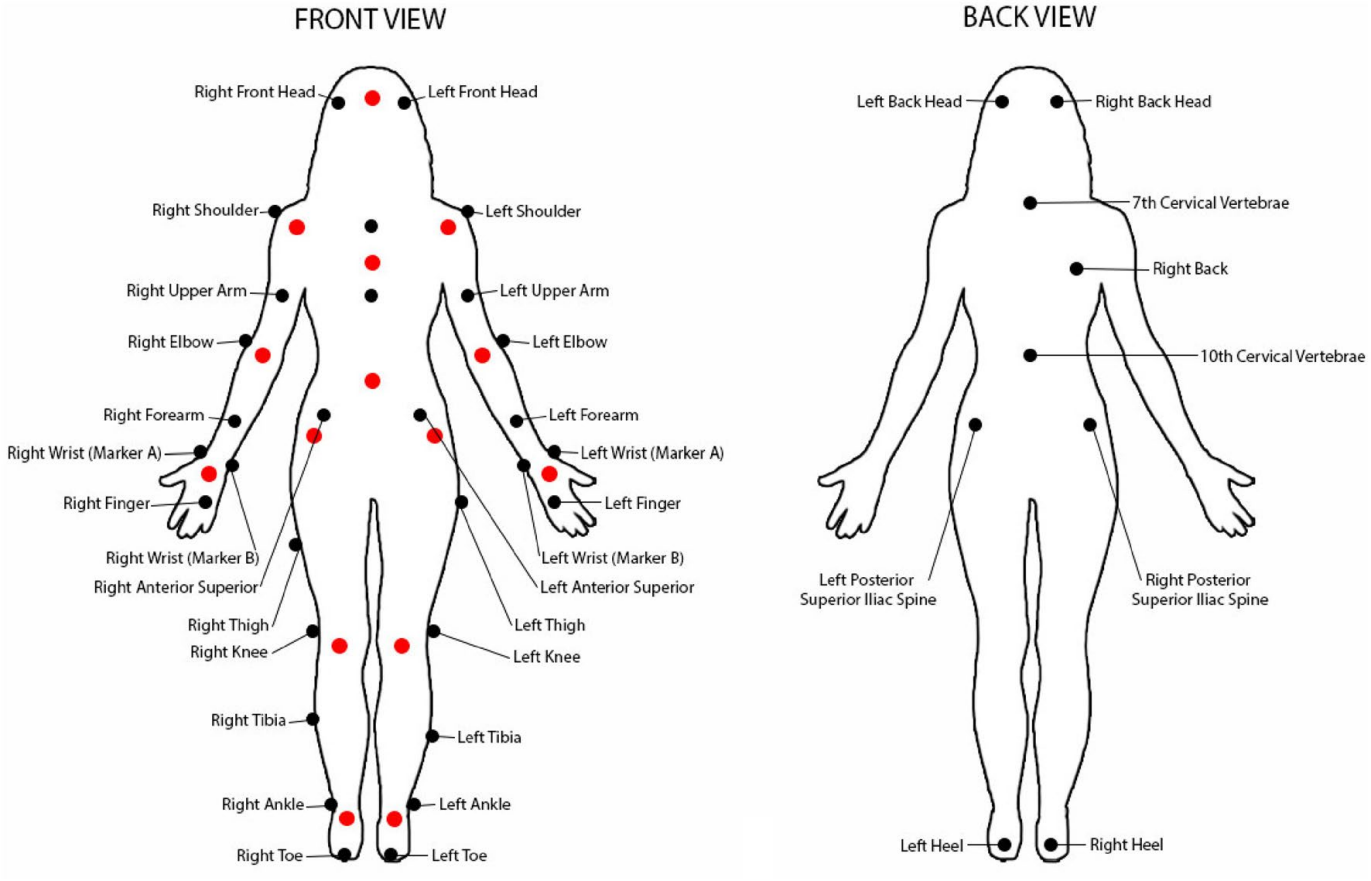
Placement of retroreflective markers on the body during the recording phase are denoted in black. The Plug-In Gait template in Vicon Nexus was used to convert the original 39 markers to point-light display figures (the final markers are denoted in red)

In the Nexus software, each marker was labelled on a frame-by-frame basis. A skeleton template was then applied over each recording to generate 15 new body markers from the original 39 placed on the dancer’s body. These 15 new markers depict a simplified figure with less visual clutter, while maintaining the overall body form (see Fig. 1 for more detail about marker transformation).

For each of the movement recordings, 3D coordinate information was extracted from the Vicon Nexus data files. Then for each time frame of each clip, the 2D coordinates depicting the upright, frontal perspective were extracted and plotted on a scatterplot (as white points on a black backdrop) to create a static point-light display image. In Python, these images were then displayed one after the other (in a manner akin to the creation of stop-motion animation) and presentation timestamps were adjusted by a factor of *0.23 (to visually match the speed of the original recording) to generate a dynamic point-light display for each sequence.

As a result of the highly dynamic nature of some of the movements and sequences, some movement information was lost during the recording process, resulting in significant gaps in the coordinate data. To overcome these issues, many ‘*gap-filling*’ techniques exist, each varying in their accuracy depending on the type of data they applied to. For simpler movement sequences (e.g., knocking, walking) a linear extrapolation (from point x, where the gap begins, to point y, where the information returns) may be sufficient but for highly dynamic, complex dance movements linear extrapolation is too simplistic, and can result in movement distortion. For this data, it was determined that a combination of gap-filling techniques should be implemented on a case-by-case basis. Two visual examples (Supplementary Figs. 1 and 2) and more detail about this gap-filling procedure are available in section S2 of the Supplementary Materials.

As mentioned in the description of this library in the previous section, for some sequences, the gaps were too large or numerous to create a complete, non-distorted point-light display clip. Therefore, 2 full movement sequences (2 sequences × 5 emotion portrayals = 10 recordings), and 2 individual recordings (1 angry, 1 fearful) were discarded at this stage, leaving 73 point-light display recordings to form the final stimuli set (15 neutral, 15 happy, 15 sad, 14 angry, 14 fearful).

## MCNORM 1: first validation experiment

### Methods

#### Open science statement

Consistent with open science practices widely adopted within psychological research (Open Science Collaboration, 2012), we report all manipulations and all measures in the study. In addition, following open science initiatives (Munafò, 2016), the data, stimuli, and analysis code associated with this study are freely available on the Open Science Framework. By making the data available, we enable others to pursue tests of alternative hypotheses, as well as more exploratory analyses.

### Participants

Previous attempts to generate and validate movement libraries have participant samples sizes that have ranged between 12 and 80 participants (please see Supplementary Table 1 for an overview). While the average number of participants tested in this prior literature is around 40 participants, we aimed to collect data from at least that many participants in our two validation experiments. 1179 participants started the validation experiment, but due to the volume of incomplete responses, only those who completed the task in full were included in the analysis. Fifty participants completed the entire task, and thus made up the final sample for the first validation experiment. Participants ranged in the amount of previous dance experience they had, but they were assigned to one of five different dance-level groups (non-dancer, beginner, intermediate, advanced, or professional) by indicating which label they thought was most applicable to their previous experiences. A more detailed breakdown of sample demographics is presented in Table 1. The experiment was created in formr (Arslan et al., 2020) and was advertised through the University of Glasgow subject pool, and on a variety of social media channels (including Twitter, Facebook, and Instagram).

### Stimuli

The 73 complete movement sequences outlined in the previous section were uploaded to Vimeo and were displayed through formr using embed codes. In Vimeo, the embed codes were manipulated to remove the standard online video handles (video title, uploader information, suggested videos, and Vimeo branding). The video controllers were also removed, and the video was set to loop automatically until participants had submitted their responses for the item. The stimuli were all presented on screen at a consistent size of 496 × 370 pixels.

### Procedure

Participants followed the formr link to begin the experiment. After reading the information and consenting to participate (in accordance with BPS guidelines), participants completed two questionnaires: a series of questions extracted from an early, unpublished, version of the Goldsmiths Dance Sophistication Index (Gold-DSI; Rose et al., 2020), and the Toronto Empathy Scale (TES; Spreng et al., 2009).

Selected questions from the early Gold-DSI included demographics measures which allowed participants to provide detail about their previous dance experience, covering both formal and informal experience, and both visual and physical experience. Participants were also asked to select which level of dance experience was most applicable to them from one of five options (non-dancer, beginner, intermediate, advanced, or professional. The version of the early Gold-DSI which was used in this experiment can be found on the OSF (https://osf.io/458sq/). The TES was used to generate a trait empathy score for each participant (ranging from 0 to a maximum of 40) and includes a variety of questions (both forward and reverse scored) covering facets of both emotional and cognitive empathy.

Testing began with a short practice block, containing five different point-light display movements, to ensure participants understood the instructions and that there were no issues with video playback. Following this, participants began the validation experiment wherein they watched each of the 73 movement sequences (presented in random order) and after observing each sequence were asked “What emotion do you think the dancer is trying to convey?” in a forced-choice paradigm (neutral, happy, sad, angry or fearful). For each clip, they also responded on a sliding scale to the questions “How intensely is the emotion being expressed?” from not intense (0) to very intense (100), and “How sure are you of your decision?” from very uncertain (0) to very certain (100). The whole experiment took between 45 and 60 min for the volunteers to complete and first-year psychology students at the University of Glasgow were given 3–4 participation credits for taking part.

As this experiment was conducted using an online sample, three catch trials were included to test whether participants were paying sufficient attention to the stimuli and task. For this purpose, a separate point-light display sequence was manually edited twice to depict two new scrambled dot motion videos. At random points throughout the duration of the experiment, these two scrambled motion videos and the original unedited version appeared and on the next screen participants were asked “Did you perceive human motion in the previous clip?”. The correct answer was only “Yes” for one of these three trials. However, in hindsight, it was decided that this question may have been too open to interpretation, as the scrambled motion videos were created from an actual motion clip and subjects may have observed, for example, an arm-like dot configuration that they perceived to be humanlike in motion. Moreover, when examining the impact of correct responses to the catch trials on task performance, no significant difference in accuracy was observed between those who passed (*M* = 26.66%) and failed (*M* = 27.13%) the attention checks: *t*(47.63) = 0.38, *p* = 0.71. Therefore, it was decided that failing to correctly respond to the attention checks would not warrant immediate exclusion of that participant from the analysis.

## Results

### Perceived emotion results

The average percentage recognition of the intended emotion, across all participants and all emotions was 26.9% (SD = 4.49). With a baseline of 20% established as chance level (from a 1 in 5 chance of selecting the intended emotion), after normality testing (*p* > 0.05), a one-sample t-test revealed that overall participants identified the intended emotions at greater than chance level: *t*(49) = 10.86, *p* < 0.001, *d* = 1.54.

**Fig. 2:**
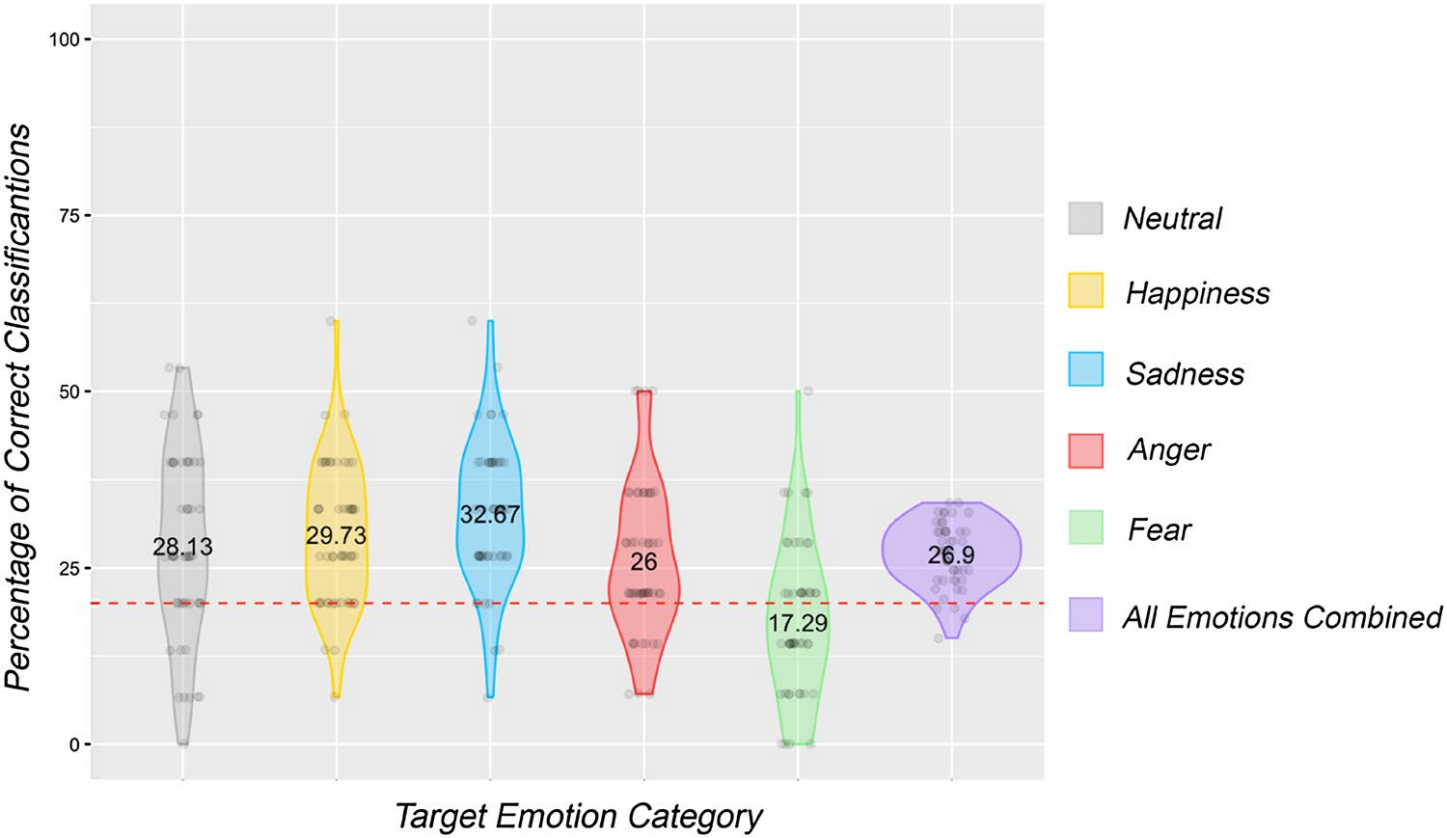
Violin plots depicting the distribution of recognition rates for each emotion category. Values presented in the centre of each of the violins represent the mean recognition rate, and the red dotted line indicates chance level of recognition. All emotions, with the exception of fear, were recognised at greater than chance level

Examining each emotion individually, it was found that all emotions, with the exception of fear, were recognised at significantly greater than chance level. See Table 2 for a summary of recognition rates. Further, the mean recognition rates for each expression category, and the distribution of responses can be observed in Fig. 2.

**Table 2:** A summary of recognition rates for each emotion category (means and standard deviations), and the results of inferential tests performed on the data to determine whether they were recognised at greater than chance level

Emotion	Normally distributed?	Average recognition rate (%)	Greater than chance?	Test	Results
Neutral	Yes	28.13% (SD = 13.21)	Yes	One-sample *t* test	*t*(49) = 4.35, p < 0.001, * d* = 0.62
Happy	No	29.73% (SD = 10.1)	Yes	One-sample Wilcoxon signed rank test	Z = 713, p < 0.001, *d* = 0.96
Sad	Yes	32.67% (SD = 10.28)	Yes	One-sample t test	*t*(49) = 8.71, p < 0.001, * d* = 1.23
Angry	No	26% (SD = 10.78)	Yes	One-sample Wilcoxon signed rank test	Z = 1023, p < 0.001, *d* = 0.56
Fearful	No	17.29% (SD = 11.18)	No	One-sample Wilcoxon signed rank test	Z = 465, p = 0.095, *d* = 0.24

Although all emotions but fear were recognised at greater than chance level, a number of frequent misclassifications still occurred. Movements intended to communicate neutral expression were often perceived to communicate happiness (26.34%). Movements intended to express anger were also mistaken for happiness (27.63%), in fact even more often that they were labelled correctly (26.47%). Fear was confused with neutrality (23.21%), happiness (21.49%), and sadness (23.21%), more often than it was correctly labelled (17.34%). A more detailed breakdown of the correct classification and misclassification rates can be found in Table 3.

**Table 3:** Correct classification and misclassification rates for each emotion category in Experiment 1

		Perceived emotion				
		Neutral	Happy	Sad	Angry	Fearful
Intended emotion	Neutral	**28.36%**	26.34%	19.49%	11.56%	14.25%
	Happy	21.10%	**29.97%**	21.1%	17.47%	10.35%
	Sad	19.41%	20.49%	**33.02%**	14.56%	12.53%
	Angry	23.45%	27.63%	10.79%	**26.47%**	11.65%
	Fearful	23.21%	21.49%	23.21%	14.76%	**17.34%**

Bold values represent the correct classification rates

These results provide support for the primary hypothesis, which was that participants should recognise the intended emotion at a level greater than chance. The data show that overall recognition accuracy across all participants was statistically greater than chance. It was also predicted that there would be variation in recognition rates for each specific emotion category, with movements expressing fear being recognised the least well. The data supports the secondary hypothesis as fear was the only emotion category not to be recognised at greater than chance level, and sadness was recognised at a higher rate than any other emotion.

The relatively high standard deviations for each emotion category suggested variation in recognition rates for each individual clip in the stimuli set. Exploratory analysis confirmed this idea, as recognition rates were found to vary widely for each individual clip in the movement library. Recognition of neutral expression varied from 4% to 62% across the 15 clips in the stimuli set. Similarly, recognition of happiness varied from 4% to 60% and recognition of anger varied from 2% to 66% from clip to clip. For both the sad and fearful clips, some stimuli were never assigned the intended emotion category (with recognition of sad ranging from 0 to 66%, and recognition of the fearful expressions ranging from 0% to 44%). See Fig. 3 in the Supplementary Materials for a detailed breakdown of recognition rates for each individual clip in the McNorm library.

### Exploratory analysis

#### Impact of dance experience on recognition

Participants completed a number of measures from an early unpublished version of the Gold-DSI to provide detail about their prior dance experience. One of these measures asked participants to classify their level of dance experience according to the following options: non-dancer, beginner, intermediate, advanced, or professional. A Kruskal–Wallis test was conducted to determine whether a participant’s self-reported level of dance experience had an impact on their ability to correctly identify the target emotion from clips in the McNorm library. However, no significant differences were observed between these groups (Chi-square = 2.597, *p* = 0.627, df = 4). It should be noted here that participants were not asked to provide numerical answers about their years of experience, the frequency of their training, or the age at which they first engaged with dance classes (in any form), so examination of these more specific aspects of physical dance experience was not possible with these data.

Participants completed a number of additional measures, three of which were related to physical dance experience (“How often do you currently go dancing for fun/social reasons?”, “I have taken regular dance classes at least once a week for…”, and “I have had formal training in any dance style for…”) and two measures were related to visual experience (“How often do you watch dance performances/shows/videos on TV or Internet?”, and “How often do you attend live dance performances?”). Therefore, these item responses were grouped together to create a more general score for each participant’s physical dance experience and observational dance experience, respectively. An additional item from the questionnaire was not related to the frequency of experience, instead asking participants to indicate the number of dance styles they had experience with (using the options; ‘none’, ‘one dance style’, ‘two dance styles’, ‘three dance styles’, or ‘more than three dance styles’) and was therefore considered separately in the analysis. As the number of possible responses to these items in this questionnaire varied from 5–7, prior to further analysis the responses were transformed using min–max normalization to account for the differences in scales.

A three-stage hierarchical multiple regression was conducted with average recognition rate as the dependent variable. The physical dance experience factor was entered at the first stage of the regression, the observational dance experience factor was added in the second stage, and number of styles a participant was familiar with was added at the final stage to create the maximal model. As this analysis is exploratory in nature, the factors were added in this order to account for the frequency with which these facets of dance experience have been explored in previous research in this field. The results can be found in Table 4.

**Table 4:** Summary of hierarchical multiple regression analysis for factors predicting recognition accuracy in McNorm Experiment 1

Model	Summary	Predictor	*Β*	*T*	SE
Model 1	*F*(1,48) = 0.367^n.s.^, *R*^2^_Adj_ = −0.013, RSE = 4.524	Intercept	27.366		
		Physical Experience	−1.162^n.s^	−0.606	1.916
Model 2	*F*(2,47) = 0.580 ^n.s.^, *R*^2^_Adj_ = −0.017, RSE = 4.534	Intercept	26.605		
		Physical experience	−2.357^n.s^	−1.006	2.343
		Observational experience	2.385^n.s^	0.891	2.678
Model 3	*F*(3,46) = 0.387 ^n.s.^, *R*^2^_Adj_ = −0.039, RSE = 4.581	Intercept	26.642		
		Physical experience	−1.965^n.s^	−0.577	3.406
		Observational experience	2.416 ^n.s^	0.891	2.712
		Number of Styles	−0.438^n.s^	−0.160	2.734

*N* = 50; *n.s*. *p* > 0.05, *p < 0.05, **p < 0.01, ***p < 0.001.

Model 1: percentage recognition − physical dance experience factor

Model 2: percentage recognition −  physical dance experience factor + observational dance experience factor

Model 3: percentage recognition − physical dance experience factor + observational dance experience factor + number of dance styles a participant has experience with

The hierarchical multiple regression revealed that at Stage one, the regression model was not a significantly better fit for the data than the null model *F*(1,48) = 0.367, *p* = 0.547, and that the physical dance experience factor only explained 1.3% of the variation in recognition accuracy. Introducing the observational dance experience factor at Stage two did not significantly improve the model when compared with the null model *F*(2,47) = 0.580, *p* = 0.563, and the observational dance factor only explained an additional 0.4% of the variation in recognition rates. Finally, the maximal model added the number of dance styles a participant had experience with to the model, but the addition of this predictor did not significantly improve the fit of the model to the data *F*(3,46) = 0.387, *p* = 0.763 and only explained an additional 2.2% of the variance in recognition rates. Therefore, previous dance experience (as measured by items from an early version of the Gold-DSI) did not have a significant impact on recognition of the target emotion in this experiment.

### Relationship between empathy and recognition of the intended emotion

Participants completed the Toronto Empathy Scale to provide a measure of trait empathy. Possible scores for this measure range between 0 and 64. A Pearson’s correlation was conducted to explore the relationship between trait empathy and recognition of the intended emotion and no significant relationship was observed: cor = 0.0048, *p* = 0.973.

### Intensity ratings

In addition to assigning an emotion category to each movement sequence, participants also provided a measure of how intensely they felt the movements portrayed their chosen emotion on a slider scale from 0 (not intense) to 100 (very intense). The average overall intensity score provided by participants, across all emotions was 56.69 (± 10.5). The average intensity scores provided for clips in each emotion category can be found in Table 5.

**Table 5:** Average intensity ratings for clips in each emotion category in Experiment 1

	Neutral	Happy	Sad	Angry	Fearful
Mean intensity score	54.36 (± 12.32)	57.44 (± 11.72)	57.54 (± 11.1)	59.02 (± 11.33)	55.18 (± 10.6)

From the mean intensity scores, it appears that portrayals of neutral expression were assigned the lowest intensity scores and portrayals of anger were assigned the highest intensity scores. A one-way ANOVA was conducted to explore the significance of these differences and no significant differences were observed in intensity scores across the different emotion categories: *F*(5,249) = 1.132, *p* = 0.34, *d* = 0.14.

### Certainty ratings

Participants also provided a measure of how certain they were of their emotion judgements on a slider scale from 0 (very uncertain) to 100 (very certain). The average certainty score for all participants across the entirety of the task was 54.12 (± 13.8). The mean certainty scores for each emotion category can be found in Table 6.

**Table 6:** Average certainty ratings for clips in each emotion category in Experiment 1

	Neutral	Happy	Sad	Angry	Fearful
Mean certainty score	52.97 (± 14.31)	55.9 (± 15.79)	53.09 (± 13.85)	55.89 (± 14.94)	52.76 (± 13.74)

Standard deviations are listed in italics within the parentheses below each average rating

It appears that participants were least certain about their categorisation of neutral and fearful expressions and most certain about their perceptions of happiness and anger. However, a one-way ANOVA revealed that these differences were not significant: *F*(5,294) = 0.51, *p* = 0.77, *d* = 0.104.

## Subset of the McNorm Library: clips with highest agreement in perception of the intended emotion

Due to the large variation in recognition rates across individual movement clips in the stimuli set, a subset of these clips was examined further. The four clips with the highest rate of agreement in emotion perceptions (i.e., the highest recognition rates for the intended emotion) across the different emotion categories were isolated for further analysis. The selected clips are highlighted in Fig. 3 of the Supplementary Materials, which also provides a more detailed breakdown of average recognition rates for these clips (and all other clips in the McNorm Library).

**Fig. 3:**
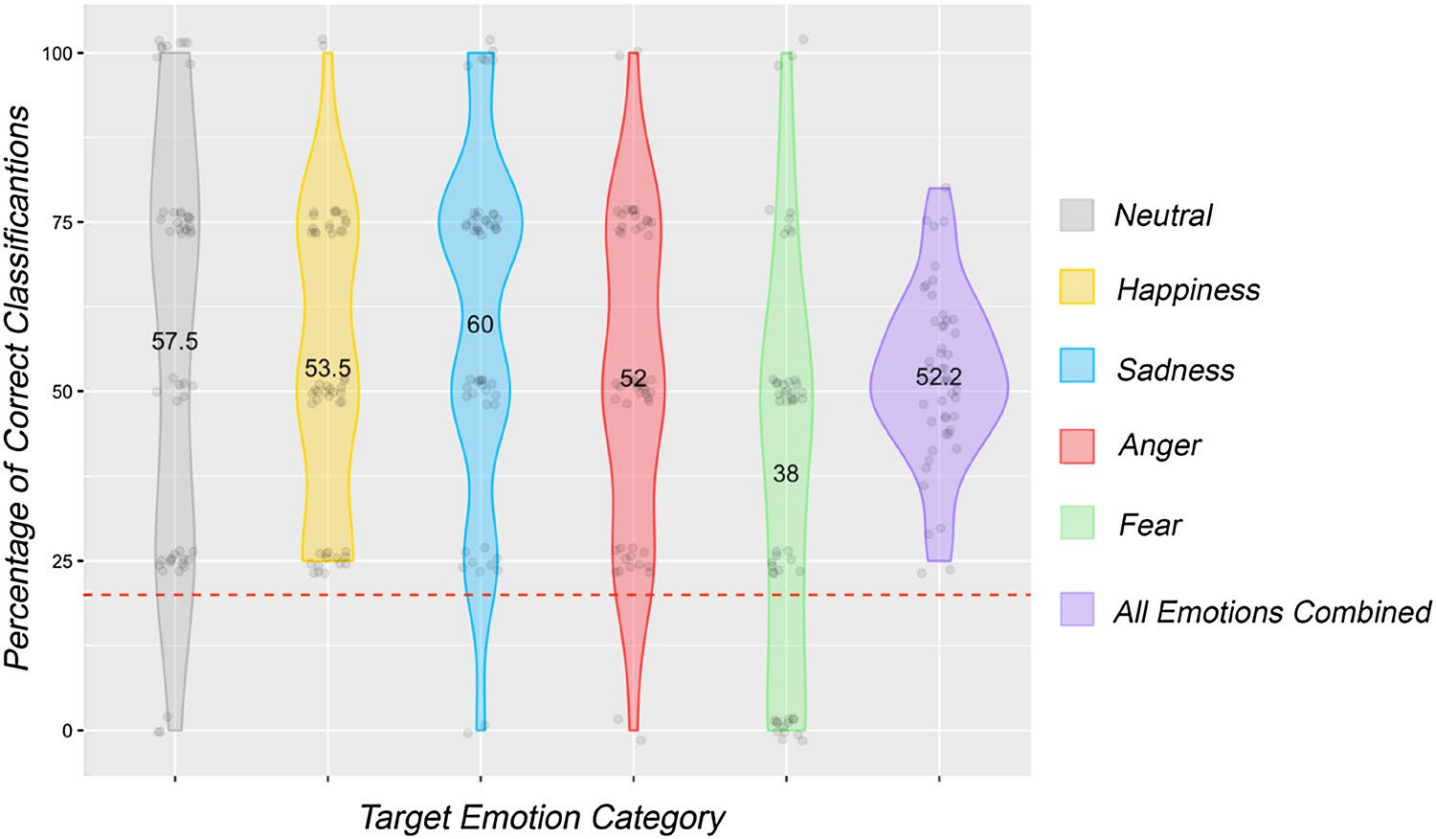
Violin plots depicting the distribution of recognition rates for each emotion category for ONLY the 20 clips from the full McNorm library identified for future study. Values presented in the centre of each of the violins represent the mean recognition rate, and the red dotted line indicates chance level of recognition. All emotion categories were recognised at greater than chance level for this subset of clips

### Recognition results

For the new subset of the McNorm stimuli, the average overall percentage recognition of the intended emotion was 52.2% (± 12.42). See Fig. 3 for a summary of recognition rates across each individual emotion category and the distribution of participants’ responses. With a baseline of 20% established as chance level, after normality testing (*p* > 0.05), a one-sample *t*-test revealed that overall participants identified the intended emotions at greater than chance level: *t*(49) = 18.33, *p* < 0.001, *d* = 2.59.

Examining each emotion individually for the new subset of clips it was found that all emotions, were recognised at significantly greater than chance level. See Table 7 below for summary of recognition rates.

**Table 7:** A summary of descriptive and inferential statistics for the subset of 20 clips from the full McNorm library identified for future study

Emotion	Normally distributed?	Average recognition rate (%)	Greater than chance?	Test	Results
Neutral	No	57.5% (SD = *30.83)*	Yes	One-sample Wilcoxon Signed Rank Test	Z = 1227, p < 0.001, *d* = 1.22
Happy	No	53.5% (SD = 22.02)	Yes	One-sample Wilcoxon Signed Rank Test	Z = 1275, p < 0.001, *d* = 1.52
Sad	No	60% (SD = 25.75)	Yes	One-sample Wilcoxon Signed Rank Test	Z = 1256, p < 0.001, *d* = 1.55
Angry	No	52% (SD = 23.6)	Yes	One-sample Wilcoxon Signed Rank Test	Z = 1248, p < 0.001, *d* = 1.36
Fearful	No	38% (SD = 29.55)	Yes	One-sample Wilcoxon Signed Rank Test	Z = 1054, p < 0.001, *d* = 0.61

While this subset of clips from the McNorm library was recognised with greater accuracy, were a number of common misclassifications persisted. Expressions of happiness were often confused for expressions of anger (27.27%) and conversely, anger was often mistaken for happiness (31.66%). Fearful expressions were often considered to depict sadness (23%) and sadness was sometimes confused with neutral expression (19.29%). Neutrality was also often perceived to be happy (16.08%). Table 8 provides a more detailed breakdown of correct classifications and misclassifications attributed to this subset of the McNorm library.

**Table 8:** Correct classification and misclassification rates for each emotion category for the subset of 20 clips from the McNorm library identified for further study

		Perceived emotion				
		Neutral	Happy	Sad	Angry	Fearful
Intended emotion	Neutral	**57.79%**	16.08%	11.56%	4.02%	10.55%
	Happy	7.07%	**54.04%**	8.59%	27.27%	3.03%
	Sad	19.29%	5.08%	**60.91%**	3.55%	11.17%
	Angry	5.53%	31.66%	4.02%	**52.26%**	6.53%
	Fearful	6.5%	14%	23%	18.5%	**38%**

Bold values represent the correct classification rates

### Intensity ratings

In addition to assigning an emotion category to each movement sequence, participants also provided a measure of intensity of expression on a slider scale from 0 (not intense) to 100 (very intense). The average overall intensity score provided by participants, across all emotions was 60.43 (± 11.3). The average intensity scores provided for clips in each emotion category can be found in Table 9.

**Table 9:** Average intensity ratings for each emotion category for the subset of 20 clips from the full McNorm movement library

	Neutral	Happy	Sad	Angry	Fearful
Mean intensity score	46.94 (± 18.02)	66.08 (± 15.61)	58.81 (± 14.59)	72 (± 12.78)	58.01 (± 14.29)

From the mean scores outlined above, it appears that participants perceived certain emotions to be portrayed with greater intensity than others in this subset of clips from the full McNorm library. A one-way ANOVA confirmed that several of these differences were significant: *F*(5,249) = 16.74, *p* < 0.001, *d* = 0.56. After correcting for multiple comparisons (with a Tukey HSD test), it was found that expressions of anger were perceived to be significantly more intense than expressions of fear (*p* < 0.001), neutrality (*p* < 0.001), and sadness (*p* < 0.001), but not more intense than happy expression (*p* = 0.33). Neutral expressions were assigned significantly lower intensity ratings than sad (*p* < 0.001), happy (*p* < 0.001) and fearful expressions (*p* < 0.005). There were no significant differences in intensity ratings for sequences intending to communicate happiness and fear (*p* = 0.07), or happiness and sadness (*p* = 0.13), or fear and sadness (*p* > 0.9). No other significant differences were observed (all p values > 0.05).

### Certainty ratings

Participants’ provided certainty on their judgements based on a sliding scale from 0 (very uncertain) to 100 (very certain) throughout the experiment. The average certainty score for all participants across the entirety of the task was 56.64 (± 14.5). The mean certainty scores for each emotion category can be found in Table 10.

**Table 10:** Average certainty ratings for each emotion category for the subset of 20 clips from the full McNorm movement library

	Neutral	Happy	Sad	Angry	Fearful
Mean certainty score	54.19 (± 18.68)	60.96 (± 16.85)	54.22 (± 16.66)	60.44 (± 17.78)	53 (± 17.67)

It appears that participants were most certain about movements in the happy and angry expression categories, and least certain about the fearful, sad and neutral expressions. However, the results of a one-way ANOVA found that these differences were not statistically significant: *F*(5,294) = 1.996, *p* = 0.08, *d* = 0.20.

### Summary

The reason for the large variation in recognition rates across all clips in the full McNorm library is unclear. A lack of attention to the task may be one potential explanation. It should be noted here that this experiment took between 45 and 60 min to complete in full, and it is well established that engaging in long duration experiments which involve the presentation of repetitive stimuli can lead to a decrease in task attention (see Langner & Eickhoff, 2013, for a comprehensive meta-analysis of these findings). Catch trials were included in an attempt to exclude participants who did not fully attend to the stimuli, however in this experiment these trials were deemed to be too subjective to use as a benchmark for participant exclusion. This may have been further exacerbated by remote data collection. In non-laboratory settings, the possible influence of environmental distractors cannot be ruled out. Therefore, as it was not possible to control for distractions, and especially given nature of this task, it is possible that low attention levels (or a high number of distractions) contributed to the relatively low recognition rates seen in Experiment 1. Alternatively, given the fairly high recognition rates for some of the movement stimuli, it is possible that the subset of clips isolated for further analysis are simply more representative in their portrayals of the intended emotions. To explore this possibility, a second validation experiment was devised and conducted using only this new subset of clips.

## McNorm 2: second validation experiment

### Methods

#### Participants

A total of 722 participants started this experiment. After excluding participants who did not complete the task in full, the final sample size was 77. A more detailed breakdown of sample demographics can be found in Table 11. The experiment was also created in formr (Arslan et al., 2020) and was advertised through the University of Glasgow subject pool, and on social media channels. Participants who participated in Experiment 1 were informed that they were not eligible to take part in Experiment 2.

**Table 11:** Demographics information for participants who took part in the second McNorm Experiment (N = 77)

Age	Mean (SD)	31.96 (13.09)
	Range	20–66
Gender	Female	66
	Male	10
	Transgender female	NA
	Transgender male	NA
	Gender variant/non-conforming	NA
	Missing	1
Dance level	Non-Dancer	24
(self-reported)	Beginner	29
	Intermediate	18
	Advanced	5
	Professional	NA

### Stimuli

The four clips with the highest recognition rates, for each emotion category (as identified in the first experiment) were used (i.e., 4 × each emotion category (5; neutral, happy, sad, angry, fearful) = 20 clips). As before, these clips were shown in formr using Vimeo embed links (with all video handles removed).

### Procedure

As in the first experiment, after reading the experiment information and providing informed consent, participants provided responses to the shortened version of the early Gold-DSI and the Toronto Empathy Scale. They then took part in a practice block with five trials (using the next best-recognised clips for each emotion category) to ensure they understood the task instructions and that there were no video playback issues. Then participants watched each of the 20 movement sequences (presented in random order) and responded to the question “What emotion do you think the dancer is trying to convey?” from the same five options (neutral, happy, sad, angry or fearful). They also provided responses on the same intensity and certainty slider scales, as participants did in experiment 1. The whole experiment took around 15 min to complete and first year psychology students at the University of Glasgow were given participation credits for their time.

As it was determined that the attention check used in the first experiment was too open to interpretation, catch trials in Experiment 2 were adjusted for greater clarity. The same three scrambled dot and normal point-light display clips were presented randomly during the experiment, but this time, participants were asked “Did you perceive a clear human form in the previous video?”. The answer “Yes” was only correct in one of these three instances. It was determined that this question was less ambiguous, and therefore a more sensitive measure of attention than the check put in place in Experiment 1. Indeed, in this instance, when examining the task performance of those who responded incorrectly to the catch trials, it was found that participants who failed to pass the attention checks (i.e., those who answered one or more of the questions incorrectly) performed significantly worse at the main task of identifying emotions (*M* = 41.74%) than participants who passed these checks (*M* = 48.96%): *t*(43.35) = −2.18, *p* = 0.035. Therefore, those who failed the attention checks (*N* = 23) in Experiment 2 were excluded from the analysis, leaving 53 participants in the final sample.

## Results

### Perceived emotion results

In the Experiment 2, the average overall percentage recognition of the intended emotion, across all 53 participants was 48.96% (SD = 13.6). With a baseline of 20% established as chance level (from a 1 in 5 chance of selecting the intended emotion), after normality testing (*p* > 0.05), a one-sample t-test revealed that overall, participants identified the intended emotions at greater than chance level: *t*(52) = 15.504, *p* < 0.001, *d* = 2.13.

**Fig. 4:**
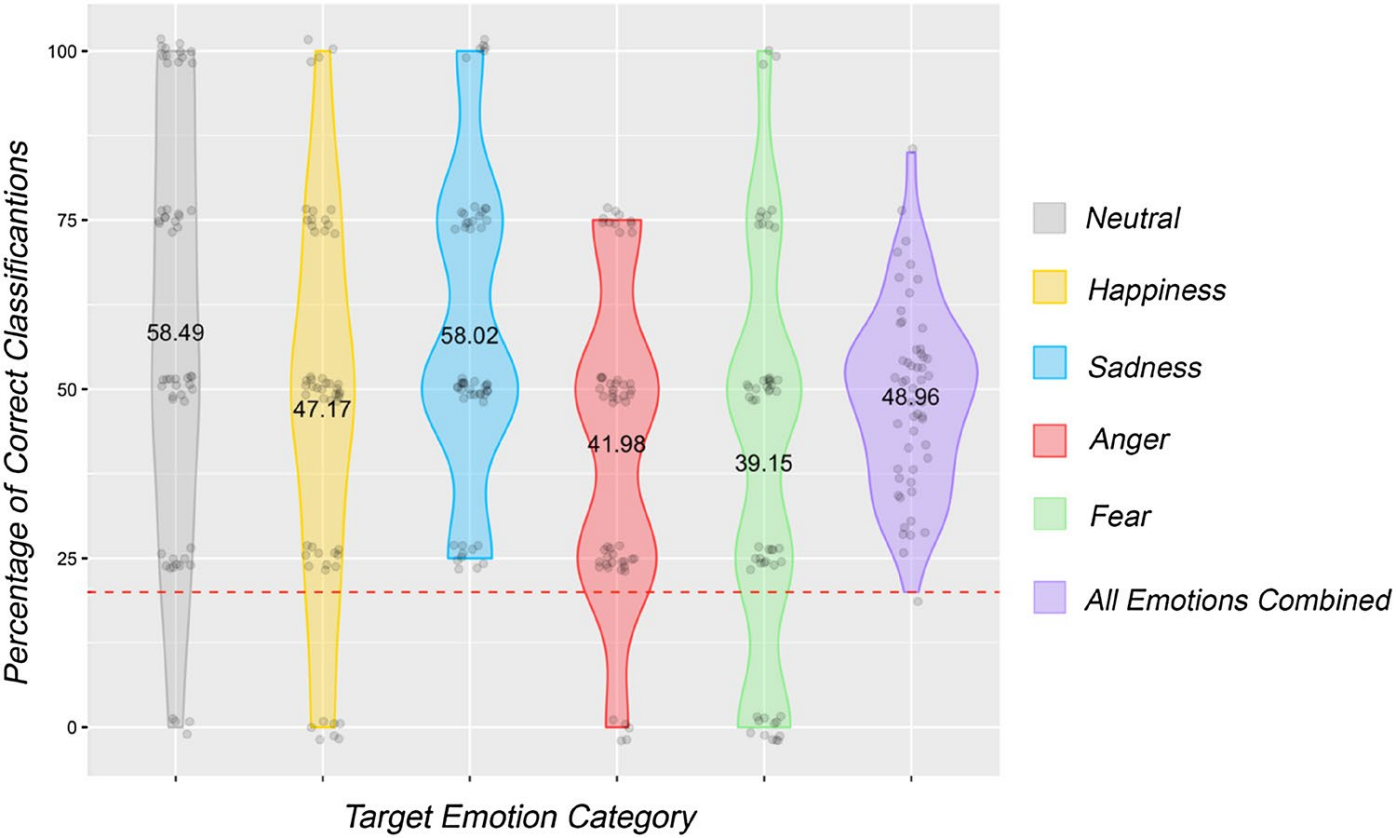
Violin plots depicting the distribution of recognition rates for each emotion category in the second experiment. Values presented in the centre of each of the violins represent the mean recognition rate, and the red dotted line indicates chance level of recognition. All emotion categories were recognised at greater than chance level

Examining each emotion individually, all intended emotion categories were recognised at a level significantly greater than chance. See Table 12 for a summary of recognition rates, and Fig. 4 for a visualisation of the responses.

**Table 12:** A summary of recognition rates for each emotion category (means and standard deviations), and the results of inferential tests performed on the data to determine whether they were recognised at greater than chance level for the second experiment

Emotion	Normally distributed?	Average recognition rate (%)	Greater than chance?	Test	Results
Neutral	No	58.49% (SD = 30.99)	Yes	One-sample Wilcoxon Signed Rank Test	*Z* = 1381, p < 0.001, *d* = 1.24
Happy	No	47.17% (SD = 28.02)	Yes	One-sample Wilcoxon Signed Rank Test	*Z* = 1326, p < 0.001, *d* = 0.97
Sad	No	58.02% (SD = 22.88)	Yes	One-sample Wilcoxon Signed Rank Test	*Z* = 1431, p < 0.001, *d* = 1.66
Angry	No	41.98% (SD = 22.88)	Yes	One-sample Wilcoxon Signed Rank Test	*Z* = 1326, p < 0.001, *d* = 0.96
Fearful	No	39.15% (SD = 29.22)	Yes	One-sample Wilcoxon Signed Rank Test	*Z* = 1197, p < 0.001, *d* = 0.66

While recognition rates in the second McNorm validation experiment were much higher than those found in the first experiment, a number of common misclassifications were still present. Participants frequently confused angry expressions with happiness (42.45%), and this occurred at a marginally greater rate than correct classification (41.98%). However, angry expressions were rarely perceived to portray any of the other emotion categories. Happy was also commonly confused with anger (24.06%), although at a lower rate than the converse. Fearful expressions were often confused with sadness in this sample (25.47%), and sad expressions were sometimes confused with fear (19.34%). A more detailed overview of classification and misclassification rates for the subset of clips from the full McNorm library can be found in Table 13.

**Table 13:** Correct classification and misclassification rates for each emotion category in Experiment 2

		Perceived emotion				
		Neutral	Happy	Sad	Angry	Fearful
Intended emotion	Neutral	**58.49%**	12.74%	11.79%	4.25%	12.74%
	Happy	15.57%	**47.17%**	8.96%	24.06%	4.25%
	Sad	13.68%	7.55%	**58.02%**	1.42%	19.34%
	Angry	8.02%	42.45%	2.83%	**41.98%**	4.72%
	Fearful	15.57%	8.96%	25.47%	10.85%	**39.15%**

Bold values represent the correct classification rates

### Summary

These results provide support for the primary hypothesis, as the intended emotion was recognised at greater than chance level for neutral, happy, sad, angry, and fearful expressions. Additional support was obtained for the secondary hypothesis, as recognition rates were found to vary across the different emotion categories; with neutral and sad expressions being recognised at the highest rate, and fearful expressions recognised with the lowest frequency.

In addition, these results suggest that the inconsistencies in recognition rates across all clips in the first McNorm validation experiment were not due to a lack of task attention, but rather, these results support the idea that stimuli clips isolated from the full library for further analysis are simply more representative of the target emotions than others in the full stimuli set.

### Exploratory analysis

#### Impact of dance experience on recognition

As in the first experiment, participants completed a number of measures from an early unpublished version of the Gold-DSI to provide detail about their prior dance experience. A Kruskal–Wallis Test was conducted to determine whether participants’ self-reported level of dance experience had an impact on recognition accuracy for the target emotions. In this experiment no participants classified themselves as a professional dancer, so there were only four levels of self-reported experience (non-dancer, beginner, intermediate, and advanced) used in this analysis. No significant differences were observed between the four groups (Chi-Square = 2.396, *p* = 0.494, df = 3).

As in the first experiment, a three-stage hierarchical multiple regression was conducted with average recognition rate as the dependent variable. The physical dance experience factor (a factor created as before from three measures in the early Gold-DSI) was entered at the first stage of the regression and the observational dance experience factor (a factor created as before from two measures in the early Gold-DSI) was added in the second stage. The number of styles a participant was familiar with was added at the final stage to create the maximal model. A detailed breakdown of the results can be found in Table 14.

**Table 14:** Summary of hierarchical multiple regression analysis for factors predicting recognition accuracy in the second experiment

Model	Summary	Predictor	*Β*	*T*	SE
Model 1	*F*(1,51) = 0.031^n.s..^, *R*^2^_Adj_ = −0.019, RSE = 13.73	Intercept	49.340		
		Physical Experience	−1.154^n.s^	−0.176	6.550
Model 2	*F*(2,50) = 4.325*, *R*^2^_Adj_ = 0.113, RSE = 12.81	Intercept	54.897		
		Physical experience	8.434^n.s^	1.217	6.928
		Observational experience	−24.626^**^	−2.935	8.391
Model 3	*F*(3,49) = 2.874*, *R*^2^_Adj_ = 0.098, RSE = 12.92	Intercept	54.974		
		Physical experience	10.214^n.s^	1.183	8.633
		Observational experience	−23.909^**^	−2.746	8.708
		Number of styles	−2.331^n.s^	−0.351	6.636

*N *= 50; *n.s*. *p* > 0.05, *p < 0.05, **p < 0.01, ***p < 0.001

Model 1: Percentage recognition − physical dance experience factor

Model 2: Percentage recognition − physical dance experience factor + observational dance experience factor

Model 3: Percentage recognition − physical dance experience factor + observational dance experience factor + number of dance styles a participant has experience with

The hierarchical multiple regression revealed that at Stage one, the regression model was not a significantly better fit for the data than the null model *F*(1,51) = 0.031, *p* = 0.861, that the physical dance experience factor was not a significant predictor of recognition accuracy, and that this factor only explained 1.9% of the variation in recognition accuracy. Introducing the observational dance experience factor at Stage two contributed significantly to the regression model *F*(2,50) = 4.325, *p* = 0.019. The observational dance factor was found to be a significant predictor of recognition accuracy (*p* = 0.005) and explained an additional 9.4% of the variation in recognition scores. Finally, the maximal model was also found to explain the data significantly better than the null model *F*(3,49) = 2.874, *p* = 0.046. However, the number of dance styles a participant had experience with was not a significant predictor of recognition accuracy, and the inclusion of this factor decreased the amount of variance the model explained by 1.5%. Therefore, it appears that the most important predictor for task performance (as measured by average recognition rate) in this experiment was the observational dance factor (i.e., the frequency with which participants watch dance on screen (TV, phone, or computer) and in live settings). Together the three variables presented in the maximal model accounted for 9.8% of the variance in recognition rates.

### Empathy results

A Spearman correlation was conducted to explore the relationship between self-reported trait empathy (as measured by responses on the Toronto Empathy Scale) and recognition of the intended emotion in this second McNorm validation experiment. No significant relationship was observed: *r*_*s*_ = **−**0.132, *p* = 0.345.

### Intensity ratings

In addition to assigning an emotion category to each movement sequence, participants also provided a measure of how intensely they felt the movements portrayed their chosen emotion on a slider scale from 0 (not intense) to 100 (very intense). The average overall intensity score provided by participants, across all emotions was 55.82 (± 11.58). The average intensity scores provided for clips in each emotion category can be found in Table 15.

**Table 15:** Average intensity ratings for clips in each emotion category in Experiment 2

	Neutral	Happy	Sad	Angry	Fearful
Mean intensity score	46.68 (± 16.65)	61.48 (± 14.06)	53.48 (± 15.16)	63.26 (± 15.13)	54.19 (± 14.04)

From the mean intensity scores, it appears that portrayals of neutral expression were assigned the lowest intensity scores and portrayals of anger were assigned the highest intensity scores. A one-way ANOVA was conducted to explore the significance of these differences and several differences were observed in intensity scores across the different emotion categories: *F*(5,318) = 9.18, *p* < 0.001, *d* = 0.40.

After correcting for multiple comparisons using a Tukey HSD test, angry expressions were perceived to be significantly more intense than fearful (*p* = 0.016), neutral (*p* < 0.001) and sad (*p* < 0.01) expressions. The difference in intensity scores for angry and happy expressions was not found to be significant (*p* = 0.99). In addition, happy expressions were perceived to be significantly more intense than expressions of neutrality (*p* < 0.001). Although movements expressing happiness were perceived to be more intense than portrayals of sadness, this difference only approached significance (*p* = 0.05). No other significant differences were observed (all *p* values > 0.05).

### Certainty ratings

Participants also provided a measure of how certain they were of their emotion judgements on a slider scale from 0 (very uncertain) to 100 (very certain). The average certainty score for all participants across the entirety of the task was 53.61 (± 15.1). The mean certainty scores for each emotion category can be found in Table 16.

**Table 16:** Average certainty ratings for clips in each emotion category in Experiment 2

	Neutral	Happy	Sad	Angry	Fearful
Mean CERTAINTY score	54.67 (± 14.18)	55.34 (± 17.2)	54.69 (± 15.67)	53.11 (± 19.17)	50.55 (± 16.99)

It appears that participants were least certain about their categorisation of fearful expressions and most certain about their perceptions of happiness. However, a one-way ANOVA revealed that these differences were not significant: F(5,318) = 0.59, *p* = 0.71, *d* = 0.12.

## General discussion

### Emotion recognition results

Results from both validation experiments showed that participants recognised the intended emotion from movements in the McNorm library at greater than chance levels. In the first experiment, the average overall recognition rate across all participants was just above chance; at 26.9%. This rate was far lower than expected based on the results of previous work in this area (Atkinson et al., 2004; Castellano et al, 2007; Crane et al., 2013; Dael et al., 2012; Gross et al., 2010; Michalak et al., 2009; Paterson et al., 2001; Roether et al., 2009; Wallbott, 1998). Our first thought was that this low recognition rate may have been the result of conducting a long duration, repetitive task in a non-laboratory setting (where environmental distractors could not be controlled for) with inadequate catch trials. However, further examination of the data revealed that this comparatively low recognition rate could perhaps be attributed to large variability in recognition rates across individual clips that made up each emotion category across the stimuli set. We suspected that some movement sequences in the full McNorm library did not convey a specific emotion as clearly and universally as others did. This idea was confirmed by the results from the second validation experiment. Experiment 2 examined responses from a new participant sample to a subset of the most well-recognised clips identified during Experiment 1. Recognition rates obtained in Experiment 2 were more in line with those reported in previous work, with an average recognition rate across all participants of 48.96%.

In addition to validating the McNorm library as a tool for social communication research, this result could be important to consider more broadly in the creation of future motion capture libraries. It is likely that when generating large samples of expressive movement data, there will be some specific sequences or movement clips that more effectively communicate the intended emotion better than others. These experiments present a novel solution to handling the occurrence of stimuli which do not perform as intended in the exploration of a particular research question. Perhaps in the future, researchers should examine their stimuli set as a whole and, if some individual (stimuli) do not serve their intended purpose, they could consider condensing the number of stimuli to create a new subset which can be exposed to further testing. This paradigm may result in more reliable materials for human movement research.

As predicted, based on recognition rates reported in the wider emotion recognition literature, fear was consistently recognised at the lowest rate. There are several relevant theories to discuss, and a wealth of potential explanations for this finding that would merit future investigation. For one, while the classic James–Lange, Cannon–Bard, and Schacter and Singer theories disagree about the specific mechanisms of the experience of emotions, these perspectives are all based on the core principle that physiology and emotions are intrinsically linked (Bard, 1928; Cannon, 1927; James, 1884; Lange, 1885; Schachter & Singer, 1962). The fear response, in particular, appears to be highly embodied; causing a uniquely recognizable set of physical manifestations within the human body. For many years, a fight-or-flight response was widely accepted as the dominant model for behavioural response to environmental stressors (Cannon, 1927). However, more recently, Barlow described an adaptive alarm model which includes, not only fight and flight, but also freeze as a potential response (Barlow, 2004; Schmidt et al., 2008). Therefore, even at the most basic physiological level, responses to fear-inducing triggers are highly idiosyncratic. It should be acknowledged that in creating the McNorm library, the method for portraying fear was decided solely by the movement performer. It is possible that this dancer’s singular perspective was insufficient to capture the full scope of how fear manifests through movement, and may be different to how observers themselves experience fear—resulting in the lower levels of recognition. It would be useful for future work to examine the idiosyncrasies of fearful movement from the perspective of multiple dancers.

In addition, returning to the classic theories of emotion mentioned above, it is worth noting that they all indicate the need for some sort of trigger, or stressor, to produce the physiological arousal and elicitation of emotions (whichever order these responses appear in). It is possible that in the absence of an emotional trigger, this resulted in the dancer producing less authentic displays of the target emotions during the creation of the McNorm library. Results from facial expression research provide evidence that observers can differentiate between spontaneous (Duchenne) and posed (non-Duchenne) smiles (Etcoff et al., 2021; Krumhuber & Manstead, 2009; Trutoiu et al., 2014). Additional research in this area has found that fear is one emotion which observers are particularly sensitive to; in detecting inauthenticity from facial expressions (McLellan et al., 2010). However, to our knowledge, no research has explored observer sensitivity to the authenticity of different emotions expressed through human body movement. Based on these factors, and the low recognition of fear reported here and in previous work (Atkinson et al., 2007; Camurri et al., 2003; Dahl & Friberg, 2007; Dittrich et al., 1996; Pasch & Poppe, 2007), it is possible that observers are more sensitive to detecting inauthentic fear expressions from the body in motion. To explore this idea, it could be useful in the creation of future movement libraries to implement an emotion elicitation task prior to collection of the movement data and to more closely examine the physiological responses of the performer (e.g., heart rate, galvanic skin response) to determine how authentically ‘felt’ the emotions were during the movements (Val-Calvo et al., 2020). These movement sequences could then be compared to those obtained from an alternative set of movements (generated without a prior emotion elicitation session) in an emotion recognition task to explore the impact of posed versus genuine expressivity on observer judgements of emotion. Whether fear is particularly sensitive to this effect or not, it would be beneficial to social cognition research to examine the role of authenticity in recognition of affective expression from the body across all of the basic emotions. Findings from such works could have a significant impact on methods for creating future libraries of emotionally expressive human movement stimuli. In sum, although largely speculative, these are two factors which may explain why fear was recognised with the lowest levels of accuracy in the McNorm experiments and in the wider emotional movement literature.

### Dance experience and emotion recognition results

The data from both validation experiments did not provide support for the idea that dance experience facilitates emotion recognition abilities. Kruskal–Wallis tests performed on data from each experiment found that there were no significant differences in recognition accuracy across participants with different self-reported levels of dance experience. In addition to physical experience, it was found that increased experience of observing dance did not lead to improved recognition rates. In fact, in the second experiment, observation experience loaded negatively onto the recognition accuracy factor; thus, for the sample population in experiment two, increased amounts of observational dance experience led to a decrease in average recognition accuracy score.

Previous research has consistently reported that dance experience results in changes to behavioural outcomes (Christensen et al., 2019; Stevens et al., 2010), physiological responses (Christensen et al., 2016; Kirsch et al., 2016) and brain function and well as structure (Bläsing et al., 2012; Calvo-Merino et al., 2005; Cross et al., 2006; Cross et al., 2009; Hänggi et al., 2010; Kirsch & Cross, 2015; Orlandi & Proverbio, 2019). Therefore, it is surprising that participants who reported having prior dance experience did not show higher recognition of the intended emotion in the McNorm library validation studies. However, it should be noted that in previous work, experienced dance participants were typically expert performers (recruited from professional companies). In this validation study, there were only two participants who categorised themselves as professional dancers, and both were in the sample population for Experiment 1. Therefore, the majority of dancer participants in these experiments represent an often-neglected group in this area of research: amateur dancers. As so few studies explore the impact of informal or hobby dance experience on behavioural outcomes, the role of amateur experience on behavioural factors, like emotion recognition capabilities, remains unclear. It is possible that amateur dance training, regardless of duration, is not sufficient to facilitate the improved emotion recognition capabilities seen in expert performers, and this may explain the results obtained from the McNorm Experiments (at least in part). However, until future work specifically examines the role of non-professional dance training, and how this differs to professional training, this interpretation remains speculative.

Further, the role of observational dance experience on emotion recognition accuracy is unclear. While the results from the second McNorm experiment suggests that observational experience has a negative effect on recognition of the target emotion, this result should be interpreted carefully as this effect was not observed in the first experiment. Based on the previous literature and the lack of generalizability across the McNorm experiments, it is likely that this finding was specific to the sample population recruited for Experiment 2. There are a number of potential explanations for this result. First, it should be acknowledged that data for Experiment 2 was collected during the Covid-19 pandemic. The observational experience measures from the version of the Gold-DSI used in these experiments related exclusively to the frequency with which participants engaged with dance performances. During national lockdowns which took place across the globe, access to dance performances was extremely limited, and it was not clearly explained that responses to the Gold-DSI should reflect participants’ behaviour during normal circumstances. Therefore, it is possible that participants were under-reporting the frequency with which they would engage with dance performances in pre- (or non-) Covid times, and this may have undermined the usefulness of these results. On this note, it is likely that questions relating purely to the frequency of attending performances do not fully reflect engagement with dance as an artform. There are a number of factors that may impact engagement beyond simply attendance, such as enjoyment, emotional response, and the extent to which watching dance makes the observer want to move themselves. Many of these elements of engagement can be examined using the published version of the Gold-DSI (Rose et al., 2020; which was unavailable at the time of data collection for the McNorm experiments), therefore future research should make use of the full questionnaire, and of other more general aesthetic engagement questionnaires (e.g., the Aesthetic Experience Questionnaire; Wanzer et al., 2020, and the Aesthetic Responsiveness Assessment; Scholtz et al., 2020), to examine more completely what an individual may gain from engaging with art, and dance specifically, as a spectator, and how this may influence social behaviours.

### Empathy results

Several studies have reported a relationship between individual differences in empathy and recognition of affective information. This has been observed in identification of emotion from facial expressions, voices, and body movement (Besel & Yuille, 2010; Fridenson-Hayo et al., 2016; Golan et al., 2007). However, across both experiments of this study, we found no evidence of a relationship between empathy scores and recognition of the intended emotion. In this study, we used the Toronto Empathy Scale to generate a measure of trait empathy for each participant. This measure is composed of 16 items and covers several aspects of the empathic response in everyday scenarios. However, it is possible that the reduced format of this scale did not produce a robust enough measure of empathy. In this study, the inclusion of a more detailed empathy measure may have been detrimental to the results. The first validation study, which contained all 73 items of the full McNorm library, was around 45 min in duration and required a high level of cognitive demand (due to the presentation of highly repetitive stimuli). Therefore, it was decided that a short measure of empathy should be included to limit interference of participant fatigue on the results. However, in future work it may be more useful to include a more detailed measure of empathy to explore this relationship further.

### Intensity and certainty ratings

Although no significant differences in certainty scores emerged across the different expression categories, participants tended to report they were most certain of their judgements when assigning emotion labels to portrayals of happiness and anger. It is interesting to note here that participants showed the highest accuracy in recognition of sad expressions in the first study, and the greatest levels of recognition for neutrality and sadness in the second study. In addition, when examining misclassification rates across both studies, it was found that participants frequently confused happy and angry expressions with one another. It is possible that participants believed it was easier to assign expression labels to more intense emotion categories. The data do provide support for this assumption, as happy and angry expressions were perceived to communicate emotion with the greatest intensity, compared to sadness, fear and neutrality. Unsurprisingly, portrayals of neutral emotion were assigned the lowest intensity ratings. However, it is surprising that portrayals of neutrality received an intensity score of roughly 50 (on a scale of 0–100, denoting the spectrum from *not intense* to *very intense*). Neutrality in this study was described to participants as the absence of expression of any specific emotion. Therefore, it is unclear why participants did not provide an average intensity score closer to zero when asked to respond to the question of: “How intensely is the emotion being expressed?”.

The performance of certain dance styles (classical ballet in particular) is designed to communicate a narrative or meaning to observers, and this often relies on the ability to arouse emotion from an audience. In the absence of other social cues like verbal communication, and facial expressions (which audience members at the back of a dance venue may find difficult, or impossible, to perceive), the dynamic body is the dancer’s main tool for communicating expression, and formal dance training responds to this need by teaching dance students how to imbue the performance of whole-body movements with emotionally salient information. A professional ballet dancer was recruited to generate the movement sequences in the McNorm library, and as a result of her extensive experience in performative dance it was likely that the dancer found it difficult to create and perform truly neutral movement sequences. This is likely a problem for all dance-based movement libraries, as creating and performing non-expressive choreography directly contradicts training and defies the communicative nature of performative dance. This may explain why participants assigned surprisingly high intensity ratings to the neutral expressions in this set of validation studies and is an issue worth addressing when creating future dance movement libraries.

### Benefits of the McNorm library

In creating the McNorm library, several methodological issues from previous work were identified and addressed. First, the McNorm library contains movements which depict expression of four basic emotions (happy, sad, angry, and fearful) in addition to neutral, or non-expressive, movements. Recent findings from Jack and colleagues suggest that there are only four universally recognised facial expressions (‘happy’, ‘sad’, ‘surprise/fear’, and ‘disgust/anger’) (Jack et al., 2016). However, it is currently unclear whether this framework for recognition of facial expressions applies to expression through the human body in motion. To begin answering this question, it would be beneficial for researchers working in this area to expand their research questions to explore perceptions of more than positive versus negative valence, to first tackle the basic, universal emotions (e.g., happiness, sadness, anger, fear) before exploring more socio-culturally complex emotions. This approach would result in greater methodological consistency and would improve the ease with which results can be compared across studies in this domain.

Further, the McNorm library addresses the question of how emotion is portrayed through whole-body movement while accounting for both parties involved in the dyadic nature of expressive communication (i.e., the movement performer and the movement observer). This approach confers significant advantages to prior movement libraries which focus exclusively on perceptions of observers when assigning movements to expressive categories.

In addition to the benefits addressed above, the McNorm library is particularly well suited to use in visual attention research. Previous movement libraries have a number of issues which impose unique limitations on their utility in visual attention experiments; depending on the research question. For example, several movement libraries address the limitations highlighted in the previous section (e.g., see work by Shafir and colleagues, who present an elegant series of papers which account for the intricacies of the movement observer-movement performer dyad) and are undeniably useful for social perception research. However, few libraries of this nature depict the desired movements in the form of point-light displays. It has been noted in previous visual attention research that a number of visual cues can influence perceptual and cognitive processing. Different levels of luminance, for example, have been found to specifically impair biological motion perception (Burton et al., 2016). This finding is of particular relevance to the present authors, and to other researchers exploring emotion perception from human movement. Therefore, in this regard, point-light displays (like those in the McNorm Library) confer a significant advantage over full-light displays for research questions related to visual attention, as the process of creating such stimuli allows for greater control over the visual features of the display. In addition to these low-level cues, higher order superficial cues can also significantly influence perceptual processing. For example, it has been suggested that our attentional processes have evolved to favour attractive stimuli (Dixson et al., 2014) and sexual interest has been found to drive visual attention and influence fixation patterns when observing human figures (Hall et al., 2011; Heron-Delaney et al., 2013). Although in the majority of full-light display movement libraries the face is obscured, and clothing and background can be simplified and held constant across portrayals, point-light displays (which are devoid of these features and of further morphological information about the performer) present an easier to use, and more appropriate stimulus for visual attention research.

Finally, the McNorm stimuli set confers one further advantage over previous movement libraries. In the first experiment, for the full stimuli set we reported a high miss-rate for recognition of some emotion categories. However, when placing these results in the context of the previous literature, the subsample of movement sequences validated across Experiments 1 and 2 present a competitive recognition rate. Based on previous emotion recognition and stimuli validation experiments, average recognition accuracy scores ranged from 59.98% to 64.95% (depending on the emotion category). A more detailed breakdown of these calculations and the data used can be found in Tables 1 and 2 of the supplementary materials. The average recognition rates obtained for the McNorm Library for these same emotion categories ranged from 38.58% to 59.01%. While average hit-rates for the McNorm Library are comparatively lower, it is worth noting that the average values obtained from our results have been calculated from two different participant samples. This signifies an advantage of the McNorm Library, as other libraries are rarely (if ever) examined for test–retest reliability. Therefore, the hit-rate for recognition is not considerably reduced in comparison with existing movement libraries, and the successful test–retest values obtained across both participant samples has generated a set of stimuli that reliably invoke perceptions of the target emotions.

As such, we believe the McNorm Library presents a reliable and useful tool for the field of social perception.

### Limitations of the McNorm library

One limitation of the McNorm library is that the portrayals of each emotion category were based on the interpretation of only one dancer. Based on the type of training (i.e., style of dance, school of dance) and cultural factors there may be variations in how dancers choose to communicate different emotions through their movements. In addition, while the dancer aimed to communicate consistent portrayals of each specific expression category, it is unclear the extent to which she perceived her performance to communicate that emotion and only that emotion. It has already been noted that prior dance experience has a significant impact on a number of behavioural and neuropsychological outcomes, and therefore, it is possible that unique experiences of the dancer used to create this movement library may have influenced various elements of her performance. For example, there may have been some specific movements within the sequences that the dancer personally associated with a specific emotion or mental state and this may have coloured the performance of movements with a different intended expression. To limit the impact of subjective interpretations in creating movement stimuli that are representative of emotional expressions, future movement libraries of this nature should include recordings of more than one dancer.

Finally, the McNorm library contains sequences derived from only two types of western dance (ballet and contemporary dance). Therefore, it is unlikely that these results can be applied to different, more abstract forms of western dance (e.g., modern dance, jazz) and certainly may not be applicable to non-western dance; given the previously discussed studies which emphasise cultural differences in the identification and communication of emotional expression (Archer, 1997; Jack et al., 2009; Jack et al., 2012a; Jack et al., 2012b; Van Dyck et al., 2017).

### Using the McNorm library in future research

Both versions of the McNorm library, (the full, original sample, and the subsample tested further in experiment 2) have a specific utility for researchers depending on the nature of the research question to be explored. For future experiments focused on emotion recognition, specifically, the present authors advise making use of only the subset library (the 20 clips subjected to further analysis in Experiment 2) rather than the full McNorm Library, as (per its intended purpose) this sample of movement clips has been identified as the most representative of the target emotions; from both a perceptual and performative perspective. However, the full stimuli set will be useful to researchers who wish to access a larger amount of data to test other hypotheses. For example, original sample would be suitable for use in biological motion and visual aesthetics research, and will also be useful for expanding the volume of data available to researchers who wish to explore the communicative value of movement through machine learning. As such, the full McNorm Library (and all 3D-position data) is also available, and can be found on the OSF (https://osf.io/458sq/).

For this validation study, the movement recordings were converted into PLD videos to explore the relationship between movement intention and movement perception, but the raw coordinate data could be used in future studies to examine the interplay of quantifiable kinematic movement parameters and observer perceptions (in emotion recognition, among other constructs). Indeed, this question is currently being examined in a separate experiment by the authors of this paper; the results of which will be shared in a future manuscript. Furthermore, dance is gaining attention in aesthetics research. In this validation study, no measure of aesthetic judgements were collected. This makes the McNorm library, in its current state, unsuitable for exploring how emotion expression contributes to the aesthetic value of dance movement. However, there are plans to rectify this issue in future work by introducing measures of *beauty*, *interest,* and/or *liking* to the experimental procedure. This will allow these PLD movement sequences to be used in future research which explores the impact of performative elements of movement on emotional recognition and aesthetic evaluation.

Creation of the McNorm library is also part of a larger scale project that will be conducted by the authors of this paper. The overall aim of this project is to create a framework for the expression of emotion to others in our social environment through dynamics of the human body in motion. It is hoped that the principles of this framework could be used to inform choreographic practices (to allow dance-makers to create movement sequences which are known to inspire expressive states in observers) and could be used by dancers in their performances (by providing direction about which performative elements of motion are most important for communicating expressivity or an emotionally charged narrative to audiences). Finally, beyond the scope of aesthetics and performative dance, it is also hoped that these principles can be applied to the field of artificial intelligence. For example, a more detailed understanding how humans communicate emotion to others may help technicians, designers, and computer scientists to create more expressive (and thus likeable) artificial interaction partners (such as robots and virtual/augmented reality avatars) which move, and ultimately behave, in a more anthropomorphic manner.

## Supplementary Information

Below is the link to the electronic supplementary material.

Supplementary file1 (DOCX 308 KB)

## Data Availability

All datasets, code and materials are freely available on the Open Science Framework (https://osf.io/458sq/).
