# Supplementary material for: The McNorm library: creating and validating a new library of emotionally expressive whole body dance movements
Source: Psychol Res. 2022 Apr 6;87(2):484–508. doi: 10.1007/s00426-022-01669-9 (PMC8985749; doi:10.1007/s00426-022-01669-9)
Supplement: Supplementary file 1 — Supplementary file1 (DOCX 308 KB) [file 426_2022_1669_MOESM1_ESM.docx]

**Supplementary Information**

*S1. SUMMARY OF PREVIOUS EMOTION RECOGNITION AND MOVEMENT LIBRARY VALIDATION EXPERIMENTS*

**Supplementary Table 1:** A brief overview of the methods and results of previous emotion recognition, and stimulus library validation experiments. This table includes the following details: the authors and date of publication, the sample size used in the experiment, the nature of the participants (e.g., university students, dancers), the type of movements explored (e.g., dance, walking - and where possible whether gestures were included or excluded), the duration of the stimuli, the way the body was represented in the stimuli (e.g., full-light displays, point-light displays, avatars), the method for collecting and measuring emotion recognition, the emotions explored in the experiment, and recognition rates reported in the papers. It should be noted that recognition rates (expressed as percentage) were not always available. Some authors used different measures (e.g., Crane & Gross (2013) used mean decoding accuracy expressed as a decimal value; Christensen et al., (2016), (2019) and (2021) used a 0-100 slider scale for the extent of expressivity, and Dahl & Friberg (2007) used a 6-point Likert scale to measure the extent of expressivity of each specific emotion), and some authors did not provide emotion-specific recognition rates (e.g., Atkinson, Tunstall & Dittrich (2007) show recognition rates for each emotion in Figure 2, but the specific values were not available in the text).

| Authors | Sample Size | Nature of Participants | Movement Type | Stimuli Duration | Experimental Design | Chance Level Recognition | Stimuli Type | Emotion Category | Recognition Rate |
| --- | --- | --- | --- | --- | --- | --- | --- | --- | --- |
| *Atkinson, Dittrich, Gemmell & Young (2004)* | N = 36 | University Students | Free Movement *(including gestural cues)* | 6 seconds | Forced-choice emotion recognition task | 20% | Point-Light Videos & Full-Light Videos | *Point-Light Videos*  Anger  Disgust  Fear  Happiness  Sadness  *Full-Light Videos*  Anger  Disgust  Fear  Happiness  Sadness | 71.94%  63.06%^■^  79.72%  84.17%^▲^  82.22%  85.55%  75.28%^■^  91.11%^▲^  86.67%  86.94% |
| *Crane & Gross (2007)* | N = 60 | University Students *(who performed the walking motions)* | Walking motions after emotion elicitation phase | Various durations | Binary choice for agreement with the felt emotion (agreement or disagreement) | 50% | Point-Light Videos | Neutral  Sad  Content  Joy  Anger | 83%^▲^  76%  74%  67%  62%^■^ |
| *Crane & Gross (2013)* | N = 42 | University Students *(who performed the walking motions)* | Walking motions after emotion elicitation phase | Various durations | Forced-choice emotion recognition task | .10 | Full-Light Videos | Anger  Content  Joy  Neutral  Sad | .23^■^  .23^■^  .24  .25  .43^▲^  *(mean decoding accuracies)* |
| *Gross, Crane & Fredrickson (2012)* | N= 30 | University Students | Walking motions | Various durations | Forced-choice emotion recognition task | 10% | Full-Light Videos | Anger  Joy  Sad  Content  Neutral | 22%  20%  43%^▲^  19%^■^  25% |
| *Montepare, Koff, Zaitchik & Albert (1999)* | N = 41 | University Students  *(N = 20)* & Older Adults *(N = 21)* | Dramatized depictions of emotions *(including gestural cues)* | 3 seconds | Forced-choice emotion recognition task | 25% | Full-Light Videos | *University Students*  Neutral  Happy  Angry  Sad  *Older Adults*  Neutral  Happy  Angry  Sad | 95.74%^▲^  73.68%^■^  90.99%  82.41%  92.34%^▲^  71.92%  78.66%  68.77%^■^ |
| *Pasch & Poppe (2007)* | N = 48 | University Students & Staff | Static Images of Body Postures | N/A | Forced-choice emotion recognition task | 16.67% | Full-Light 3D Renderings of Human Bodies and Human-Like Wooden Avatars | *Human Body Renderings*  Anger  Disgust  Fear  Happiness  Sadness  Surprise  *Wooden Avatars*  Anger  Disgust  Fear  Happiness  Sadness  Surprise | 32%  9%^■^  36%  57%  58%^▲^  32%  39%  24%  23%  62%^▲^  54%  16%^■^ |
| *Bernhardt & Robinson (2007)* | N/A | Machine Learning Algorithm | Everyday Motions *(e.g., knocking, throwing, lifting)* | Various durations | Machine Learning Algorithm based on Movement Kinematics | 25% | Point-Light Displays | *Using Unbiased Features*  Neutral  Happy  Angry  Sad  *Using Biased Features*  Neutral  Happy  Angry  Sad | 37.9%^■^  41.1%  59.1%  62.4%^▲^  74.2%  65.3%^■^  92.4%^▲^  92.4%^▲^ |
| *Dael, Mortillaro & Scherer (2012)* | N/A | Algorithm based on Movement Characteristics | Emotions Portrayed in Scenario-Based Interaction Settings by Actors | Various Durations | Algorithm based on Movement Characteristics | 8.33% | Full-Light Videos | Amusement  Pride  Elated Joy  Panic Fear  Anxiety  Despair  Sadness  Hot Anger  Irritation  Interest  Pleasure  Relief | 70%  50%  90%^▲^  60%  50%  40%  30%^■^  70%  30%^■^  60%  60%  50% |
| *Roether, Omlor, Christensen & Giese (2009)* | N = 21 | University Students | Walking motion | Various Durations | Forced-choice emotion recognition task | 25% | 3D Avatar Renderings | Anger  Happiness  Neutral  Fear  Sadness | 76.0%  65.1%^■^  71.5%  80.0%  92.0%^▲^ |
| *Gross, Crane & Fredrickson (2010)* | N = 35 | University Students | Knocking motion after emotion elicitation phase | Various durations | Intensity scores for each emotion including the target emotion on a 5-point Likert scale (classified as recognised if the target emotion was rated ‘moderately’ (3) or above in intensity | N/A | Full-Light Videos | *Across All Clips (Emotions Felt and Unfelt)*  Angry  Anxious  Sad  Joyful  Proud  Content  *Across Only Clips where Emotion was Felt*  Angry  Anxious  Content  Joy  Proud  Sad | 78%^▲^  65%  56%  39%  56%  33%^■^  8%^▲^  6%  3%^■^  3%^■^  6%  6% |
| *Camurri, Lagerl**öf & Volpe (2003)* | N = 32 | University Students | Modern Dance movements *(specifically excluding gestures)* | Various durations | Forced-choice emotion recognition task & Intensity Ratings for All Emotions | 25% | Full-Light Videos | *Across All Participants and Portrayals*  Anger  Joy  Fear  Grief | 60.6%  55.0%  39.8%^■^  70.4%^▲^ |
| *Melzer, Shafir & Tsachor (2019)* | N = 62 | General Population | Free movement *(including gestural cues)* | 3 seconds | Forced-choice emotion recognition task | 20.7%  (for the entire sample) | Full-Light Videos | Happy  Sadness  Fear  Anger  Neutral | 81.39%^▲^  78.57%  51.15%  47.29%^■^  67.42% |
| *Alaerts et al., (2011)* | N = 32 (after technical issues) | University Students | Everyday Motions *(e.g., walking, jumping, kicking)* | 3 seconds | Forced-choice emotion recognition task (happier’, ‘sadder, ‘angrier’ or ‘not-different’ to a neutral clip) | 25% | Point-Light Videos | Neutral  Happy  Sad  Angry | 54.3%  44.2%^■^  45.8%  58.6%^▲^ |
| *Atkinson, Tunstall & Dittrich (2007)* | N = 32 | University Students | Free movement *(including gestural cues)* | 3 seconds | Forced-choice emotion recognition task | 16.67% | Full-Light Videos & Point-Light Videos | *Full-Light Videos*  Overall  *Point-Light Videos*  Overall  *Note: No means for emotion-specific recognition rates were available in the paper | 84.2%  81.7%  From Figure 2, the general order of recognition rates (from highest to lowest) was  1. Sadness^▲^  2. Happiness  3. Neutral  4. Anger  5. Fear  6. Disgust^■^ |
| *Bachmann, Zabicki, Munzert & Kr*üger (2020) | N = 30 | General Population | Emotions Portrayed in Interaction Settings by Actors | 4 seconds | Forced-choice emotion recognition task | 20% | Point-Light Videos | *Full-Body Stimuli*  Overall  Anger  Happiness  Sadness  Affection  *Arms-Only Stimuli*  Overall  *Trunk-Only Stimuli*  Overall | 80.7%  87.8%^▲^  85.6%  82.1%  67.6%^■^  68.9%  57.8% |
| *Christensen et al., (2016)* | N = 44 | University Students *(N = 24)* & Ballet Dancers *(N = 20)* | Ballet Dance Movements | 5-6 seconds | Slider Scale Response from ‘Very Sad’ (0) to ‘Very Happy’ (100) | Slider Scale Value for Perfect Recognition of Happy = 100  Slider Scale Value for Perfect Recognition of Sad = 0 | Full-Light Videos | Happy  Sad | 60.54  39.68 |
| *Christensen, Azevedo & Tsakiris (2021)* | N = 41 | General Population *(with varying levels of prior dance experience)* | Western Ballet and Contemporary Dance Movements *(Emotionally Expressive- Positive, Emotionally Expressive- Negative & Non-Expressive)* | 6-8 seconds | Slider Scale Response from ‘Not at all (expressive)’ (0) to ‘Very (expressive)’ (100) | Slider Scale Value for Perfect Recognition of Expression = 100  Slider Scale Value for Perfect Recognition of Non-Expression = 0 | Full-Light Videos | Expressive Contemporary  Not-Expressive Contemporary  Expressive Ballet  Not-Expressive Ballet | 61.88  43.34  57.22  46.40 |
| *Christensen, Lambrechts & Tsakiris (2019)* | N=80 (in each experiment) |  | Western Ballet and Contemporary Dance Movements *(Emotionally Expressive- Positive, Emotionally Expressive- Negative & Non-Expressive)* | 6 seconds | Slider Scale Response from ‘Not at all (expressive)’ (0) to ‘Very (expressive)’ (100) | Slider Scale Value for Perfect Recognition of Expression = 100  Slider Scale Value for Perfect Recognition of Non-Expression = 0 | Full-Light Videos | Expressive-Positive Contemporary  Expressive-Negative Contemporary  Not-Expressive Contemporary  Expressive-Positive Ballet  Expressive-Negative Ballet  Not-Expressive Ballet | 57.99  56.16  50.46  58.49  56.51  46.22 |
| *Dittrich, Troscianko, Lea & Morgan (1996)* | N = 72 | University Students | Emotional Dance Movements | 5 seconds | Rating Questionnaire (numbers assigned to emotions based on likelihood of being the intended emotion) | Rescaled values range from 0 (least likely) to 5 (most likely) where 5 is Perfect Recognition of the Intended Emotion  Chance = 2.5 | Full-Light Videos & Point-Light Videos | *Full-Light Videos*  Surprise  Fear  Anger  Disgust  Grief  Joy  *Point-Light Videos*  Surprise  Fear  Anger  Disgust  Grief  Joy | 4.80^▲^  3.92  4.43  3.50^■^  4.65  4.79  3.11  3.02^■^  3.06  2.77  3.72^▲^  3.69 |
| *Grèzes, Pichon & de Gelder (2007)*  *[Pilot Study]* | N = 12 | Information Unavailable | Emotions Portrayed in Scenarios by Actors | 3 seconds | Forced-choice emotion Recognition Task | 33.33% | Full-Light Videos | Fear  Neutral | 79%^■^  88%^▲^ |
| *Dahl & Friberg (2007)* | N = 20 | University Students | Musician Playing the Marimba with Different Emotion Expressions | 30-50 seconds | Rating the Expression of Each Emotion on a 6-Point Scale from ‘Nothing’ (0) to “Very much’ (6) | N/A | Full-Light Videos | Happy  Sad  Angry  Fearful  *Note: No exact values for emotion-specific recognition were available in the paper | ~ 4.0^▲^  ~ 4.5  ~ 3.5  ~ 2.0^■^ |

Where ^■^ denotes the emotion with the lowest level of recognition, and where ^▲^ denotes the emotion with the highest level of recognition.

**Supplementary Table 2:** A summary of average recognition rates (%) for different types of stimuli reported in the previous emotion recognition tasks (from the publications summarised in Supplementary Table 1). Recognition rates expressed as percentage were not always available.

| **Stimuli Type** | **Emotion Category** | **Average**  **Recognition Rate (*%*)** | **Number of Data Points Included in Calculation** | **Range in Reported Recognition Rates (*%*)** |
| --- | --- | --- | --- | --- |
| **Avatar** | **Overall** | **54.78** | **11** | **16 – 92** |
| Avatar | Neutral | 71.5 | 1 | N/A |
| Avatar | Happy | 63.55 | 2 | 62 – 65.1 |
| Avatar | Sad | 73 | 2 | 54 – 92 |
| Avatar | Anger | 57.5 | 2 | 39 – 76 |
| Avatar | Fear | 51.5 | 2 | 23 – 80 |
| Avatar | Other | 20 | 2 | 16 – 24 |
| **FLD** | **Overall** | **50.67** | **64** | **3 – 95.74** |
| FLD | Neutral | 65.58 | 6 | 25 – 95.74 |
| FLD | Happy | 54.7 | 11 | 3 – 90 |
| FLD | Sad | 55.26 | 12 | 6 – 86.94 |
| FLD | Anger | 54.19 | 12 | 8 – 90.99 |
| FLD | Fear | 59.51 | 6 | 36 – 91.11 |
| FLD | Other | 38.74 | 18 | 3 – 75.28 |
| **PLD** | **Overall** | **69** | **26** | **37.9 – 92.4** |
| PLD | Neutral | 62.35 | 4 | 37.9 – 83 |
| PLD | Happy | 64.56 | 6 | 41.1 – 85.6 |
| PLD | Sad | 73.49 | 6 | 45.8 – 92.4 |
| PLD | Anger | 71.97 | 6 | 58.6 – 92.4 |
| PLD | Fear | 79.72 | 1 | N/A |
| PLD | Other | 68.22 | 3 | 63.06 – 74 |
| **Overall** | **Neutral** | **64.95** | **11** | **25 – 95.74** |
| **Overall** | **Happy** | **58.74** | **19** | **3 – 90** |
| **Overall** | **Sad** | **62.5** | **20** | **6 – 92.4** |
| **Overall** | **Anger** | **60.15** | **19** | **8 – 92.4** |
| **Overall** | **Fear** | **59.98** | **9** | **23 – 91.11** |
| **Overall** | **Other** | **40.95** | **23** | **3 – 75.28** |

*S2. MOTION CAPTURE GAP-FILLING TECHNIQUE*

For each instance of missing data, the 100 preceding and succeeding points were graphed to show the intended trajectory of the coordinates across each time point of the gap. If the gap appeared to show a linear trajectory, the data was interpolated linearly (Supplementary Figure 1). If the gap in the trajectory appeared to be parabolic then a different method was used. For seemingly parabolic gaps, the data was extrapolated linearly from the datapoints preceding the gap, and from the datapoints following the gap, the point where these lines intersected was used as the peak of the parabola and the missing data was extrapolated accordingly (Supplementary Figure 2).


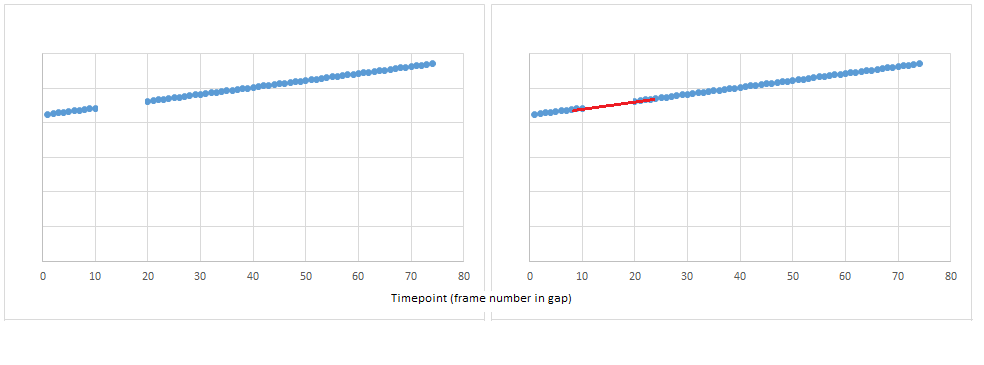


**Supplementary Fig 1:** Case-by-Case Linear Extrapolation: Shows an example of missing data which appears to follow a linear trajectory. The red line depicts the points which were extrapolated to fill this type of gap.


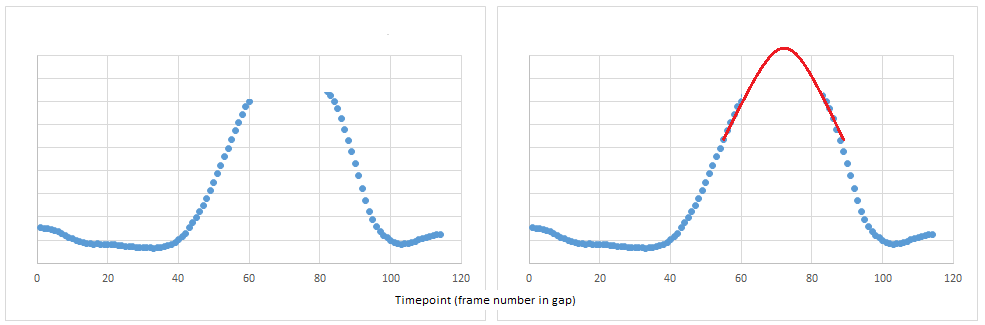


**Supplementary Fig 2:** Depicts an example of missing data which appears to follow a parabolic trajectory. On each side of the gap a linear extrapolation was carried out. The point where these two lines intersected was identified as the peak of the parabola and the surrounding points were manually smoothed to create a curve in the trajectory.

This was identified as the best method for estimating the missing data points when examining the visual output of these manipulations (compared to linear extrapolation and other such methods), but it should be acknowledged that this case-by-case method may have led to several errors that should be considered when running a computational kinematic analysis on this data. Movements contained in these sequences were often mathematically unpredictable, and the trajectories created using this method may not have accurately captured all of the complexities of this motion. However, for the creation of these point light displays, the aim was only to create stimuli with no missing movement information (i.e., to avoid markers disappearing throughout the duration of the sequence) and to minimise the amount of movement perturbation. Therefore, for this purpose, this method of gap filling was deemed sufficient.

*S3. SUMMARY OF INDIVIDUAL STIMULI IN THE MCNORM LIBRARY*

**Supplementary Table 3:** Information about each individual clip within the full, original McNorm Library.

| Movement Sequence | Emotion Portrayal | Stimuli Code | Validated in Experiment 1? | Validated in Experiment 2? | Duration  *(seconds)* | Video Size *(pixel width x pixel height)* | Average Recognition Accuracy (*%*) in Experiment 1 | Average Recognition Accuracy (*%*) in Experiment 2 | Average Recognition Accuracy (*%*) Across Experiments 1 & 2 |
| --- | --- | --- | --- | --- | --- | --- | --- | --- | --- |
| rdej | Neutral | N1 | Y | N | 31.6 | 496 x 370 | 42 | **—** | **—** |
| rdej | Happy | H1 | Y | N | 30.6 | 496 x 370 | 38 | **—** | **—** |
| rdej | Sad | S2 | Y | Y | 32.6 | 496 x 370 | 52 | 45.28 | 48.64 |
| rdej | Angry | A1 | Y | N | 30.2 | 496 x 370 | 12 | **—** | **—** |
| rdej | Fearful | F1 | Y | N | 30.4 | 496 x 370 | 14 | **—** | **—** |
| tendu | Neutral | N2 | Y | Y | 22.3 | 496 x 370 | 62 | 60.38 | 61.19 |
| tendu | Happy | H2 | Y | N | 23.0 | 496 x 370 | 32 | **—** | **—** |
| tendu | Sad | S3 | Y | N | 23.8 | 496 x 370 | 42 | **—** | **—** |
| tendu | Angry | A2 | Y | N | 21.5 | 496 x 370 | 2 | **—** | **—** |
| tendu | Fearful | F2 | Y | N | 22.6 | 496 x 370 | 20 | **—** | **—** |
| ever_con | Neutral | N3 | Y | N | 12.6 | 496 x 370 | 20 | **—** | **—** |
| ever_con | Happy | H3 | Y | N | 13.6 | 496 x 370 | 22 | **—** | **—** |
| ever_con | Sad | S4 | Y | N | 16.4 | 496 x 370 | 26 | **—** | **—** |
| ever_con | Angry | **—** | N | N | **—** | **—** | **—** | **—** | **—** |
| ever_con | Fearful | F3 | Y | Y | 17.2 | 496 x 370 | 42 | 45.28 | 43.64 |
| bitter | Neutral | N4 | Y | N | 17.1 | 496 x 370 | 6 | **—** | **—** |
| bitter | Happy | H4 | Y | N | 17.1 | 496 x 370 | 18 | **—** | **—** |
| bitter | Sad | S5 | Y | N | 17.7 | 496 x 370 | 18 | **—** | **—** |
| bitter | Angry | A3 | Y | Y | 17.4 | 496 x 370 | 52 | 35.85 | 43.93 |
| bitter | Fearful | F4 | Y | Y | 18.2 | 496 x 370 | 30 | 30.19 | 30.10 |
| char | Neutral | N6 | Y | N | 6.6 | 496 x 370 | 4 | **—** | **—** |
| char | Happy | H5 | Y | Y | 8.1 | 496 x 370 | 50 | 56.60 | 53.3 |
| char | Sad | S6 | Y | N | 9.2 | 496 x 370 | 2 | **—** | **—** |
| char | Angry | A4 | Y | Y | 7.3 | 496 x 370 | 66 | 64.23 | 65.12 |
| char | Fearful | F5 | Y | N | 7.8 | 496 x 370 | 0 | **—** | **—** |
| jete | Neutral | N5 | Y | Y | 19.9 | 496 x 370 | 58 | 58.49 | 58.25 |
| jete | Happy | H6 | Y | N | 20.9 | 496 x 370 | 42 | **—** |  |
| jete | Sad | S7 | Y | N | 20.2 | 496 x 370 | 18 | **—** | **—** |
| jete | Angry | A5 | Y | N | 19.4 | 496 x 370 | 24 | **—** | **—** |
| jete | Fearful | F6 | Y | N | 18.8 | 496 x 370 | 10 | **—** | **—** |
| fondu | Neutral | N7 | Y | N | 37.2 | 496 x 370 | 20 | **—** | **—** |
| fondu | Happy | H7 | Y | N | 42.0 | 496 x 370 | 12 | **—** | **—** |
| fondu | Sad | S8 | Y | Y | 37.3 | 496 x 370 | 68 | 67.92 | 67.96 |
| fondu | Angry | A6 | Y | N | 37.2 | 496 x 370 | 8 | **—** | **—** |
| fondu | Fearful | F7 | Y | N | 42.8 | 496 x 370 | 16 | **—** | **—** |
| free_mov | Neutral | N8 | Y | N | 26.1 | 496 x 370 | 18 | **—** | **—** |
| free_mov | Happy | H8 | Y | N | 27.9 | 496 x 370 | 14 | **—** | **—** |
| free_mov | Sad | S9 | Y | Y | 36.8 | 496 x 370 | 56 | 54.72 | 55.36 |
| free_mov | Angry | A7 | Y | N | 25.1 | 496 x 370 | 32 | **—** | **—** |
| free_mov | Fearful | F8 | Y | N | 28.1 | 496 x 370 | 18 | **—** | **—** |
| frappe | Neutral | N9 | Y | Y | 16.3 | 496 x 370 | 52 | 52.83 | 52.42 |
| frappe | Happy | H9 | Y | N | 15.4 | 496 x 370 | 22 | **—** | **—** |
| frappe | Sad | S10 | Y | N | 16.4 | 496 x 370 | 10 | **—** | **—** |
| frappe | Angry | A8 | Y | N | 15.0 | 496 x 370 | 18 | **—** | **—** |
| frappe | Fearful | F9 | Y | N | 15.8 | 496 x 370 | 6 | **—** | **—** |
| ice | Neutral | N10 | Y | N | 25.3 | 496 x 370 | 4 | **—** | **—** |
| ice | Happy | H10 | Y | N | 22.0 | 496 x 370 | 12 | **—** | **—** |
| ice | Sad | S11 | Y | N | 27.2 | 496 x 370 | 46 | **—** | **—** |
| ice | Angry | A9 | Y | N | 19.8 | 496 x 370 | 32 | **—** | **—** |
| ice | Fearful | F10 | Y | Y | 26.4 | 496 x 370 | 36 | 50.94 | 43.47 |
| balanch | Neutral | N11 | Y | N | 8.2 | 496 x 370 | 4 | **—** | **—** |
| balanch | Happy | H11 | Y | Y | 8.2 | 496 x 370 | 48 | 47.17 | 47.59 |
| balanch | Sad | S12 | Y | N | 10.9 | 496 x 370 | 0 | **—** | **—** |
| balanch | Angry | A10 | Y | Y | 10.1 | 496 x 370 | 48 | 37.74 | 42.87 |
| balanch | Fearful | F11 | Y | N | 9.4 | 496 x 370 | 0 | **—** | **—** |
| onegin | Neutral | N12 | Y | N | 24.6 | 496 x 370 | 20 | **—** | **—** |
| onegin | Happy | H12 | Y | Y | 25.7 | 496 x 370 | 56 | 43.40 | 49.7 |
| onegin | Sad | S13 | Y | N | 31.1 | 496 x 370 | 22 | **—** | **—** |
| onegin | Angry | A11 | Y | Y | 22.6 | 496 x 370 | 42 | 28.30 | 35.15 |
| onegin | Fearful | F12 | Y | N | 24.4 | 496 x 370 | 2 | **—** | **—** |
| pirouette | Neutral | N13 | Y | N | 15.4 | 496 x 370 | 34 | **—** | **—** |
| pirouette | Happy | H13 | Y | Y | 20.2 | 496 x 370 | 60 | 41.51 | 50.76 |
| pirouette | Sad | S14 | Y | N | 19.9 | 496 x 370 | 26 | **—** | **—** |
| pirouette | Angry | A12 | Y | N | 18.4 | 496 x 370 | 8 | **—** | **—** |
| pirouette | Fearful |  | N | N | **—** | **—** | **—** | **—** | **—** |
| partner | Neutral | N14 | Y | N | 23.4 | 496 x 370 | 20 | **—** | **—** |
| partner | Happy | H14 | Y | N | 23.0 | 496 x 370 | 4 | **—** | **—** |
| partner | Sad | S15 | Y | N | 28.9 | 496 x 370 | 40 | **—** | **—** |
| partner | Angry | A13 | Y | N | 20.4 | 496 x 370 | 18 | **—** | **—** |
| partner | Fearful | F13 | Y | Y | 24.1 | 496 x 370 | 44 | 30.19 | 37.095 |
| plie | Neutral | N15 | Y | Y | 29.6 | 496 x 370 | 58 | 62.26 | 60.13 |
| plie | Happy | H15 | Y | N | 30.6 | 496 x 370 | 16 | **—** | **—** |
| plie | Sad | S1 | Y | Y | 35.7 | 496 x 370 | 64 | 64.15 | 64.08 |
| plie | Angry | A14 | Y | N | 24.4 | 496 x 370 | 6 | **—** | **—** |
| plie | Fearful | F14 | Y | N | 28.4 | 496 x 370 | 4 | **—** | **—** |

**Supplementary Table 4:** A summary of the average durations and recognition rates for clips from each emotion category in the McNorm Library.

| Clip Emotion Category | Mean Duration *(seconds)* | Minimum Duration *(seconds)* | Maximum Duration *(seconds)* | Mean Recognition Accuracy (*%*) Across All Clips in the Full Stimuli Set from Experiment 1 | Mean Recognition Accuracy (*%*) for Subset of Clips Across Experiment 1 & 2 |
| --- | --- | --- | --- | --- | --- |
| Neutral | 21.08 | 6.6 | 37.2 | 28.13 *(N = 15)* | 58.00 *(N = 4)* |
| Happy | 21.89 | 8.1 | 42 | 29.73 *(N = 15)* | 50.34 *(N = 4)* |
| Sad | 24.27 | 9.2 | 37.3 | 32.67 *(N = 15)* | 59.01 *(N = 4)* |
| Angry | 20.63 | 7.3 | 37.2 | 26.00 *(N = 14)* | 46.77 *(N = 4)* |
| Fearful | 22.46 | 7.8 | 42.8 | 17.29 *(N = 14)* | 38.58 *(N = 4)* |

*S4: SUMMARY OF INDIVIDUAL RECOGNITION RATES ACROSS ALL CLIPS IN THE FULL, ORIGINAL MCNORM LIBRARY*

**
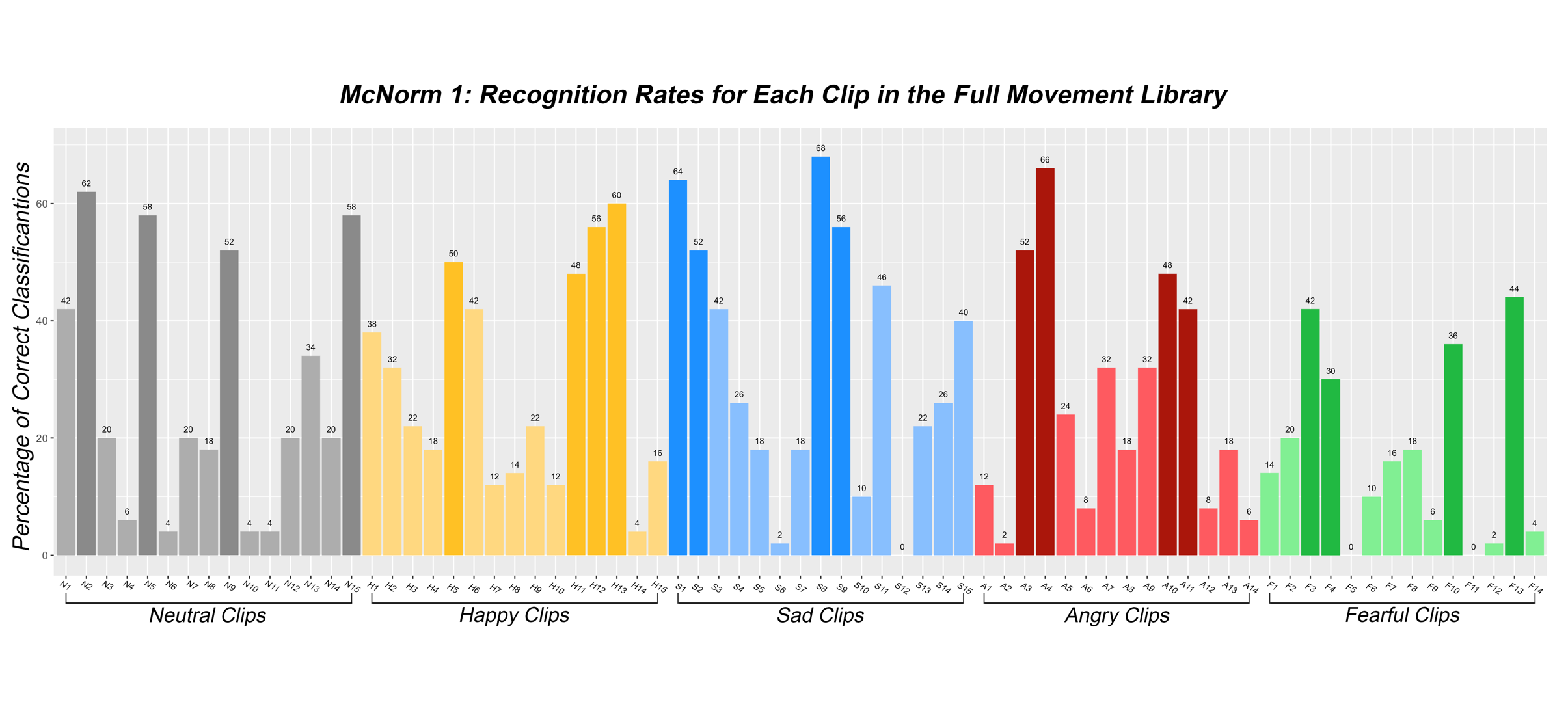
Supplementary Fig 3:** The bar plot shows average recognition of the intended emotion for each individual clip in the full McNorm Library from Experiment 1. This ranged from 0% (S12, F5, F11) to 68% (S8). Neutral clips are shown in grey, happy clips are shown in yellow, sad clips are shown in blue, anger in red, and fear in green. Highlighted bars indicate clips from the full McNorm library which were selected for further study in Experiment 2. 4 clips from each emotion category (with the highest recognition rates for the target emotion) were selected. These individual stimuli clips are N2, N5, N9, and N15 for neutral (grey); H5, H11, H12 and H13 for happy (yellow); S1, S2, S8 and S9 for sad (blue); A3, A4, A10 and A11 for anger (red); F3, F4, F10 and F13 for fear (green).
